# A mechanism for increased sensitivity of acute myeloid leukemia to mitotoxic drugs

**DOI:** 10.1038/s41419-019-1851-3

**Published:** 2019-08-13

**Authors:** Svetlana B. Panina, Natalia Baran, Fabio H. Brasil da Costa, Marina Konopleva, Natalia V. Kirienko

**Affiliations:** 1 Department of BioSciences, Rice University, Houston, TX USA; 2 Department of Leukemia, The University of Texas MD Anderson Cancer Center, Houston, TX USA; 3 Department of Diagnostics and Biomedical Sciences, The University of Texas Health Science Center, Houston, TX USA

**Keywords:** Cancer metabolism, Mitochondria

## Abstract

Mitochondria play a central and multifunctional role in the progression of tumorigenesis. Although many recent studies have demonstrated correlations between mitochondrial function and genetic makeup or originating tissue, it remains unclear why some cancers are more susceptible to mitocans (anticancer drugs that target mitochondrial function to mediate part or all of their effect). Moreover, fundamental questions of efficacy and mechanism of action in various tumor types stubbornly remain. Here we demonstrate that cancer type is a significant predictor of tumor response to mitocan treatment, and that acute myeloid leukemias (AML) show an increased sensitivity to these drugs. We determined that AML cells display particular defects in mitochondrial metabolism that underlie their sensitivity to mitocan treatment. Furthermore, we demonstrated that combinatorial treatment with a mitocan (CCCP) and a glycolytic inhibitor (2-deoxyglucose) has substantial synergy in AML cells, including primary cells from patients with AML. Our results show that mitocans, either alone or in combination with a glycolytic inhibitor, display anti-leukemia effects in doses much lower than needed to induce toxicity against normal blood cells, indicating that mitochondria may be an effective and selective therapeutic target.

## Introduction

Tumor cells exhibit a range of metabolic adaptations, including aberrant mitochondrial metabolism, abnormal expression of metabolic enzymes, and increased dependence on glycolysis for ATP generation^1,2^. They also frequently exhibit dysregulation in other mitochondrial parameters, including deregulated mtDNA content, increased ROS production, and defects in oxidative phosphorylation, suggesting that these alterations can be indicative of carcinogenesis^3–5^. Because of these defects, scientists have developed a diverse group of mitochondria-targeted anticancer drugs, called mitocans, which activate the intrinsic pathways of apoptosis and autophagy in cancer cells^6–8^. Despite this, fundamental questions remain about the utility of mitocan therapy and what mechanisms underlie susceptibility to these drugs^9^.

To identify tumor types susceptible to mitochondrial damage, we leveraged the NCI-60 panel of human tumor cell lines^10^. Using computational methods and in vitro drug toxicity assays, we identified leukemias as being particularly sensitive to mitocan treatment. A variety of mitochondrial biology assays showed a connection between respiratory coupling efficiency and mitocan sensitivity. This resulted in mitocan treatment triggering caspase-dependent cell death pathways, most likely apoptosis; we also showed that some leukemia cell lines utilize autophagy to resist this effect. Finally, we demonstrated synergy between a mitochondrial uncoupler and a glycolytic inhibitor in leukemia cells and primary AML cells, which was strongly specific for leukemia cells compared with healthy PBMCs, offering a promising therapeutic window.

## Materials and methods

### Analysis of NCI-60 panel

Two groups of drugs, mitocans and compounds not reported as targeting mitochondria (PubMed search up to January, 2018), were chosen from ~300 FDA-approved compounds screened for anticancer activity by NCI-60 project (Supplementary Table 1). For each cancer cell line/drug pair we retrieved activity *Z*-scores, representing relative sensitivity of cell line to this drug. For every compound, its activity *Z*-scores were integrated and downloaded using CellMiner^TM^ Analysis Tool (https://discover.nci.nih.gov/cellminer/)^11^. Next, we calculated sums of *Z*-scores for mitocan and nonmitocan panels of drugs per each cell line and ranked these numbers to define tumor cell lines either particularly sensitive or resistant to mitocan treatment. To compare the obtained ranks of leukemia cell lines (*n* = 6) from NCI-60 panel with random distribution, Bioconductor package ‘gtools’ was applied. Briefly, all possible combinations of 6 ranks across 1–60 ranks were permuted, following by calculation of their medians and generation of the density plot. After that, *p*-value of the difference between actual ranks and random ranks was calculated.

To determine whether cancer type affects sensitivity of tumor cell lines to treatment with mitocans or nonmitocans, multiple linear regression analysis was performed. Analysis was done with and without adjustment for age, gender, and prior treatment of the specific patient from whom the cell line was derived (patient characteristics were downloaded from Cell Line Metadata associated with CellMiner^TM^). For each factor above, B-coefficients, standard error of B, and *p*-values were calculated. The same approach was used to assess whether there was any association between prior treatment of AML patients and combination indices of drug cocktails measured in vitro using their primary samples.

### Cell cultures and primary samples

U251, a cell line derived from a glioblastoma, was chosen as a mitocan-sensitive cell line; two cell lines derived from ovarian tumors, SKOV3 and OVCAR3, were chosen for their resistance to mitocans. MOLM-13, THP-1, OCI-AML2, and MV-4-11, which were derived from human AML tumors, and SKOV3 and OVCAR3 cell lines were purchased from ATCC (Manassas, VA, USA). U251 cells were purchased from Sigma-Aldrich (St. Louis, MO, USA). A complete list of cell lines studied is available in Supplementary Table 2. Peripheral blood/bone marrow samples from patients with AML were collected during standard diagnostic procedures after informed consent was obtained in accordance with the Institutional Review Board (IRB) regulations of MD Anderson Cancer Center. The study design adhered to the tenets of the Declaration of Helsinki and was approved by the ethics committees of the participating institutions before its initiation.

Basic characteristics of the patients from whom samples were derived (including age, gender, genetic rearrangements, etc.) are shown in Supplementary Table 3. All experiments with blood cells were approved by Rice University Ethics Board Committee.

Peripheral blood mononuclear cells (PBMCs) were isolated from peripheral blood donations from either healthy blood donors (*n* = 7) or patients with AML using Leukosep tubes (Sigma-Aldrich) and Ficoll-Paque^TM^ (Sigma-Aldrich) following the manufacturer’s instructions. For all experiments, healthy PBMCs were used either shortly after isolation or rested overnight after thawing. PBMCs isolated from AML patients were used fresh and were never frozen.

Leukemia cell lines were routinely cultured in RPMI-1640 media, supplemented with 2 mM l-glutamine (Sigma-Aldrich) and 10% HyClone fetal bovine serum, FBS (GE Healthcare, Pittsburgh, PA, USA) at 37 °C in a humidified 5% CO_2_ atmosphere. Primary leukemia samples and healthy PBMCs were maintained in RPMI-1640 media with 10% FBS for 3–4 days. SKOV3 and OVCAR3 cells were respectively cultured in McCoy’s 5A media (Sigma-Aldrich) with 10% FBS and RPMI-1640 media with 20% FBS and 0.01 mg/ml human recombinant insulin (Sigma-Aldrich). U251 cells were grown in MEM media (Sigma-Aldrich), supplemented with 10% FBS, 1% nonessential amino acids (Gibco, Gaithersburg, MD, USA), and 1 mM sodium pyruvate (Gibco). Penicillin and streptomycin mix (Gibco), at a final concentration of 1%, was added to the media.

### Treatments and cytotoxicity assays

Stock solutions of carbonyl cyanide *m*-chloropheny lhydrazone (CCCP, Sigma-Aldrich), mitoxantrone (MTX, Biotang, Lexington, MA, USA), doxorubicin (DOX, Ark Pharm Inc., Arlington Heights, IL, USA), cytarabine (ara-C, Accela ChemBio Ink, San Diego, CA, USA), lonidamine (Tocris, Minneapolis, MN, USA), pan-caspase inhibitor z-VAD-fmk (Apexbio Technology, Houston, TX, USA), caspase-1 inhibitor VX-765 (Apexbio Technology), and ABT-199 (ThermoFisher, Waltham, MA, USA) were dissolved in DMSO, aliquoted, and stored at −20 °C. The glycolytic inhibitor 2-deoxyglucose, 2-DG (Chem-Impex, Wood Dale, IL, USA), and autophagic inhibitor 3-methyladenine, 3-MA (AdipoGen Life Sciences, San Diego, CA, USA) were prepared as fresh solutions in serum-free RPMI-1640 media just prior to use.

For determination of cytotoxicity, cells at a final density of 5 × 10^5^ cells/ml in serum-free RPMI-1640 media were treated with one or more drugs for 24 h for each cell line. The sole exception was cytarabine, where LD50 was determined over 48 h, with a media change and fresh cytarabine added at 24 h. Drug cytotoxicity was determined in serum-free media to prevent reaction of serum components with drugs, as this has been shown to compromise the test results on cytotoxicity^12^. Survival rates were assessed using exclusion of 0.4% Trypan Blue (Gibco) using a Countess^TM^ II FL (ThermoFisher) automated cell counter. All viability rates were normalized to corresponding solvent-control wells. The DMSO concentrations in the incubation mixtures or solvent-control wells never exceeded 0.5% (v/v).

Cells used for isolation of DNA or protein, used for flow cytometry, or measurement of ATP or ROS were treated as for cytotoxicity for 24 h. Cells used to measure expression of fusion and fission genes were treated as for cytotoxicity, but were harvested after 8 h. Oxygen consumption rate (OCR, a measure of mitochondrial respiration) and extracellular acidification rate (ECAR, which is indicative of TCA cycle and glycolysis intensity) measurements and mitochondrial membrane potential flow cytometry (using JC-1 staining) were performed after brief treatment for 4 h. Treatment durations were chosen based on preliminary time-course experiments and published studies^13^. Each experiment was carried out at least three times independently, excluding combination treatment of primary AML samples, which was conducted in 1–3 replicates due to their short-term maintenance in culture.

### DNA, RNA isolation, reverse transcription, and quantitative polymerase chain reaction (qPCR) analyses

DNA was isolated from cells using phenol/chlorophorm/isoamyl alcohol (25:24:1) extraction and isopropanol precipitation. RNA was isolated using Trizol reagent and precipitated using isopropanol. 1.5 µg of total RNA was used for reverse transcription using the AzuraQuant™ cDNA Synthesis Kit (Azura, Raynham, MA, USA). To quantify relative mitochondrial DNA content in the cells, we amplified four genomic (*ACTB*, *GAPDH*, *B2M*, and *TBP*) and two mitochondrial (*ND1* and *ND4*) gene loci using PerfeCTa SYBR^®^ Green FastMix (QuantaBio, Beverly, MA, USA). To assess mitochondrial dynamics, we measured relative expression of *MFN1*, *MFN2*, *OPA1* (fusion) and *DNM1L*, *FIS1*, *MFF*, *MID49*, *MID51* (fission) genes using AzuraQuant™ Fast qPCR Mix (Azura) normalized to *ACTB* gene expression. Analysis of qPCR data was performed using the ∆∆Ct method. We defined fusion/fission ratio as average relative expression of fusion genes (*MFN1*, *MFN2*, and *OPA1*) divided by average relative expression of fission genes (*DNM1L*, *FIS1*, *MFF*, *MID49*, and *MID51*). Amplification was performed using the CFX Connect^TM^ Real-Time PCR System (BioRad, Hercules, CA, USA). Primer sequences are shown in Supplementary Table 4.

### Fluorescence microscopy

Cells were labeled with acridine orange (ThermoFisher) at a final concentration of 2 μg/ml and propidium iodide (ThermoFisher) at final concentration of 5 μg/ml for 30 min at 37 °C, then washed with PBS, and visualized using a Zeiss M2 microscope (San Diego, CA, USA) and ZEN software. Images were taken at 20x magnification.

### Flow cytometry

Samples were analyzed on a FACSCanto Flow Cytometer (Becton Dickinson, San Jose, CA, USA). Briefly, 10^6^ total cells were stained with 200 nM of MitoTracker green (MTG, ThermoFisher) for 30 min at 37 °C and washed with PBS. Green fluorescence emissions were collected immediately after staining. Median fluorescence intensity (MFI) values, corresponding to estimated mitochondrial mass, were determined after exclusion of doublets and debris, gating target cell population, and subtraction of the background fluorescence. In addition, the MitoProbe^TM^ JC-1 assay kit (ThermoFisher) was used for assessing mitochondrial membrane potential via flow cytometry according to manufacturer’s instructions. Briefly, 10^6^ cells were stained with 1 μM of JC-1 dye for 30 min at 37 °C and washed with PBS. CCCP treatment (50 μM) was used as positive control for depolarization. Compensation was performed using excitation and emission filters appropriate for Alexa Fluor 488 (FITC) and R-phycoerythrin (PE) dyes. All analyses were done using FlowJo v.10.5.3 software.

### ATP measurement

ATP levels were measured using a bioluminescence assay by the Molecular Probes^TM^ ATP determination kit (ThermoFisher) according to the manufacturer’s instructions. A Cytation 5 Cell Imaging Multi-Mode Reader (BioTek, Winooski, VT, USA) was used to measure luminescence.

### Reactive oxygen species (ROS) measurement

Cells were stained with 20 mM (1:5000) Hoechst 33342 (ThermoFisher) and 2.5 µg/ml dihydroethidium (DHE, Cayman Chemical, Ann Arbor, MI, USA) for 30 min at 37 °C. After washing with PBS, ROS level was quantified using an RFP filter set for a Cytation 5 Cell Imaging Multi-Mode Reader (BioTek). Mean fluorescence intensities of DHE and Hoechst 33342 were quantified using ZEN software.

### Bioenergetic measurements

Real-time mitochondrial function in AML cell lines, healthy PBMCs, and mitocan-resistant SKOV3 cells was assessed using the Seahorse XF Cell Mito Stress Test kit (Agilent Technologies, Santa Clara, CA, USA) on Seahorse XFe96 Extracellular Flux Analyzer (Agilent Technologies). Briefly, cells, untreated or treated with LD50 of CCCP, DOX, 2-DG, or the combination of CCCP and 2-DG (LD25), were plated on a Cell-Tak (ThermoFisher)-coated XF96 96-well microplate using XF base media supplemented with 1 mM pyruvate, 2 mM l-glutamine, 5 mM glucose. OCR measurements were recorded after port injection starting with oligomycin (1.5 µM) followed by FCCP (1 µM), and lastly, a combination of antimycin A and rotenone (0.5 µM). All measurements were normalized to the number of viable cells. Basal respiration, ATP-coupled respiration, maximal and reserve capacities, ECAR values, as well as nonmitochondrial respiration were recorded per each condition. The results were analyzed in a Seahorse Mito Stress Test Generator (Agilent Technology).

### Western blots

Cells were washed with ice-cold PBS and lysed in RIPA buffer (50 mM Tris-HCl, pH 8.0, 150 mM NaCl, 0.5% sodium deoxycholate, 0.1% SDS, 1% Triton x-100) supplemented with Pierce protease inhibitor cocktail (ThermoFisher) followed by sonication on ice and centrifugation for 15 min at 4000 × *g*. Protein concentration in supernatants were measured via Bradford assay. Typically, 25 µg of proteins was separated by SDS-PAGE, transferred to PVDF membranes (Millipore, Bedford, MA), followed by membrane blocking at room temperature for 1 h in 1% Tween-20-TBS buffer containing 3% nonfat dry milk. Membranes were incubated at 4 °C overnight with primary mouse antibodies against either ATP synthase β subunit (ThermoFisher) or actin (ab3280) (Abcam, Cambridge, MA, USA). Membranes were washed and incubated with 1:20,000 diluted goat anti-mouse secondary antibody (Abcam) for 2 h at room temperature. After washing, membranes were incubated with SuperSignal West Femto substrate (ThermoFisher) for 2 min and exposed to film (ThermoFisher).

### Statistical analysis

Statistical analyses were performed using RStudio v. 1.1.453, GraphPad Prism v.8, and SPSS software v.23. Dose-response data for every drug were analyzed by fitting dose-response models in the Bioconductor package ‘drc’, the lethal doses for each drug (LD50 and LD25) with their 95%-confidence intervals (95% CI) were calculated^14^. A ratio-based statistical procedure was performed to compare lethal doses between two cell lines for the same drug. In this ratio test, we conclude that no difference exists in LD50 if the confidence interval for the ratio of two LD50 contains 1 or the confidence interval for the log(LD50 ratio) contains 0^15^.

To assess the effectiveness of combination therapy, we defined relative potency of two compounds for each studied cell line or primary AML sample. Then we performed cytotoxicity assays using LD25 of both drugs for each cell line or primary AML sample using single fixed-ratio mixture (1:1) ray design, as well as less concentrated mixtures in the same ratio. Finally, combination indices were calculated using the ‘drc’ package available from Bioconductor, based on mixture LD25, LD50, LD75, and plotted as a function of fraction affected (FACI plots)^16^. Drug combination landscapes were visualized using the Bioconductor package ‘synergyfinder’^17^.

The Kolmogorov–Smirnoff test was used to determine if the data were normally distributed. Multiple groups were compared by ordinary or nonparametric one-way analysis of variance (ANOVA) with subsequent pairwise, post hoc tests. Two-tailed, independent sample *t*-test was used for comparing the two groups. *p* *<* 0.05 was considered as significant. Correlations between mitochondrial parameters in untreated cells were assessed using two-tailed Pearson *r* coefficients. Also, Pearson *r* coefficients were used to estimate the correlation between drug LD50 and ratio of mitochondrial parameters, e.g., the ratio of ATP level (mean doxorubicin/mean untreated) or the ratio of mtDNA content (mean doxorubicin/mean untreated) across all studied cell lines. Protein bands were quantified and compared using ImageJ software.

## Results

### Bioinformatic analysis predicts that leukemia cells are sensitive to mitocan treatment

**Fig. 1:**
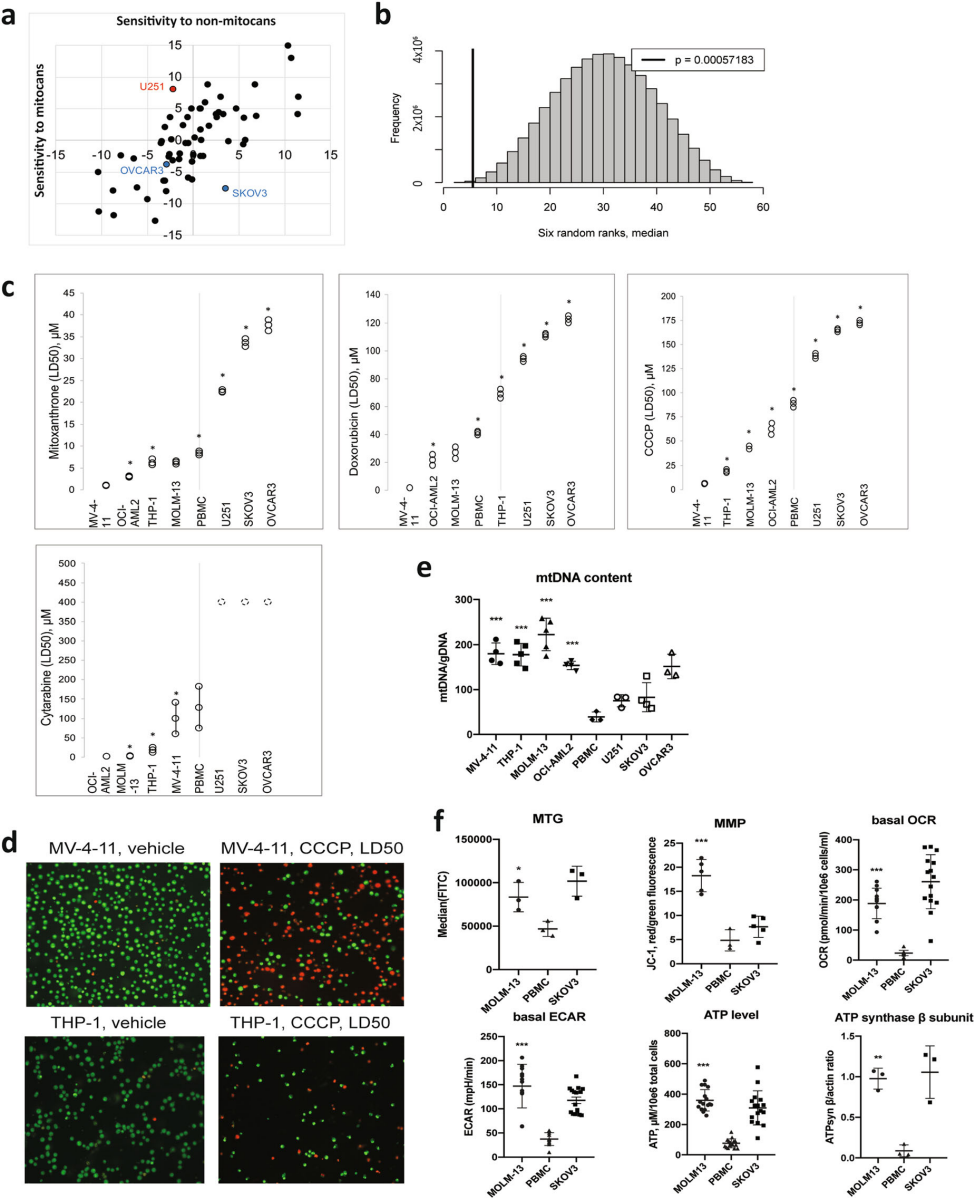
Cell lines derived from AML are more susceptible to mitochondrial damage than cell lines derived from solid tumors. **a** *Z*-scores of tumor cell lines from the NCI-60 panel. Sensitivity to non-mitocan drugs is shown on the *x*-axis, sensitivity to mitocans is shown on the *y*-axis. U251, a glioblastoma-derived cell line with high sensitivity to mitocans is shown in red, and ovarian cancer-derived cell lines, which show higher resistance, are shown in blue. **b** Density plot showing median rank for each possible permutation of six cell lines from the NCI-60 collection. The thick black line represents the median for leukemia cell lines (5.5) from the NCI-60 panel. **c** Sensitivity of AML (MV-4-11, THP-1, OCI-AML2, and MOLM-13), normal PBMCs, and solid tumor (U251, SKOV3, and OVCAR3) cell lines to mitocan treatment. Shown are LD50 values with 95% confidence intervals for MTX, DOX, CCCP, and ara-C based on results from 3–5 independent experiments. Comparisons of LD50 were done by the ratio test^15^, the asterisk indicates significant difference compared with the next most sensitive cell line. **d** Fluorescence micrographs of MV-4-11 (top) or THP-1 (bottom) cells treated with either vehicle (left) or LD50 concentrations of CCCP. Cells were stained with acridine orange/propidium iodide. **e** The ratio of mitochondrial to genomic DNA was determined by quantitative PCR. Shown are mean values with SD. Statistical significance for comparison AML vs. healthy PBMCs was analyzed via the Student’s *t*-test. **f** Mitochondrial health in untreated MOLM-13 cells and normal PBMCs were compared, including mitochondrial mass (assessed via staining with MitoTracker Green), membrane potential (assessed via staining with JC-1), metabolic rate (assessed via Seahorse analysis of oxygen consumption rate and lactate production), steady-state ATP level, and protein level of the β subunit of ATP synthase. SKOV3, a mitocan-resistant cell line, is shown as a comparison. Shown is the mean of at least three independent experiments (in case of Seahorse data and ATP measurements dots represent all technical replicates), error bars are SD. Statistical analysis was performed using Student’s *t*-test with independent samples. ***p < 0.001; **p < 0.01; *p < 0.05

To analyze the impact of mitochondrial disruption on cancer cell lines, we selected 14 molecules known to target mitochondria (mitocans) and an equal number of agents with no known mitochondrial effect (non mitocans) from a list of ~300 FDA-approved compounds screened against the NCI-60 cancer cell panel (Supplementary Table 1). Activity *Z*-scores were collected for both drug groups and summed for each cell line. The resulting number indicated the relative sensitivity of the cell line to mitocans or non mitocans compared with the overall panel (a score of 0 represented average sensitivity, positive and negative numbers showed increased sensitivity and resistance, respectively). Cell lines were then ranked by mitocan sensitivity (Table 1 and Fig. 1a).

**Table 1:** NCI-60 tumor cell lines ranked in order of decreasing sensitivity to mitocans

Rank	Sum of mitocan activity *Z*-scores	Cell line^a^
1	14.899	LE:SR
2	13.022	LE:CCRF-CEM
3	8.807	ME:LOX IMVI
4	8.805	LE:MOLT-4
5	8.142	CNS:U251
6	6.888	BR:MCF7
7	6.866	LE:HL-60(TB)
8	6.004	LC:NCI-H460
53	−7.426	CO:HCC-2998
54	−7.521	OV:SK-OV-3
55	−7.921	OV:OVCAR-4
56	−8.085	RE:TK-10
57	−9.352	OV:OVCAR-5
58	−11.273	LC:NCI-H322M
59	−11.845	ME:UACC-257
60	−12.718	OV:NCI/ADR-RES

^a^Abbreviation for tissue of origin, melanomas (ME), leukemias (LE), and cancers of breast (BR), kidney (RE), ovary (OV), prostate (PR), lung (LC), central nervous systems (CNS), and colon (CO)

Interestingly, some cancer types showed nonuniform distribution in the list. For example, leukemia cell lines clustered amongst the lines with the highest sensitivity to mitocans, while ovarian cancers displayed the opposite trend (Table 1). To further examine this observation, we compared the median rank for six leukemia cell lines included in the NCI-60 panel (SR, CCRF-CEM, MOLT-4, HL-60, RPMI-8226, and K-562, median = 5.5) to the median values for each possible combination of six cell lines (Fig. 1b). This analysis supported the conclusion that leukemia cell lines are particularly sensitive to mitocans (*p* = 5.7 × 10^−4^). Multiple linear regression analysis demonstrated a significant association between cancer type and mitocan sensitivity even after adjustment for confounds (i.e., age, gender, and the prior therapy) (Supplementary Table 5). No such association was observed for non mitocans.

### Leukemia cells are sensitive to mitocans

To validate this prediction, we obtained four acute myeloid leukemia cell lines (MOLM-13, THP-1, OCI-AML2, and MV-4-11). A glioblastoma cell line, U251, served as a mitocan-sensitive, nonleukemia cell line, and two ovarian cancer cell lines, SKOV3 and OVCAR3, were chosen for their broad-spectrum insensitivity to drugs, including mitocans. Finally, we used healthy PBMCs as a normal cell control.

We used cytotoxicity assays to derive LD50 values for four different mitocans (mitoxantrone/MTX, doxorubicin/DOX, cytarabine/ara-C, and cyanide carbonyl *m*-chlorophenyl hydrazone/CCCP) for each cell line/drug pair. Consistent with our predictions, each of the four leukemia cell lines were more sensitive to all four drugs than healthy PBMCs and ovarian cells (except the higher resistance of THP-1 cells to DOX). Surprisingly, while glioblastoma cells were more sensitive to mitocans than ovarian cells, they were more resistant than PBMCs, indicating complex, cell-type effects (Fig. 1c). We validated these results in an orthogonal assay, where MV-4-11 and THP-1 cells were treated with either vehicle or CCCP at their LD50 doses and then stained with acridine orange and propidium iodide (Fig. 1d).

To gain insights into the mechanisms underlying sensitivity, we assayed a panel of mitochondrial parameters using MOLM-13 as a representative AML cell line and healthy PBMCs as a control. Metrics tested included mitochondrial/nuclear genome ratio (mtDNA content), mitochondrial mass and membrane potential, basal oxygen consumption rate, glycolytic flux, ATP levels, and steady-state levels of the ATP synthase (Fig. 1e, f). In each case, mitochondrial activity was upregulated in MOLM-13 cells, suggesting that these cells may have a higher energy demand, as would be expected for cancerous tissue. Moreover, measurement of mtDNA content across all studied cell lines showed upregulation in every AML cell line compared with healthy PBMCs (Fig. 1e).

### Doxorubicin inhibits mitochondrial function in AML cells

**Fig. 2:**
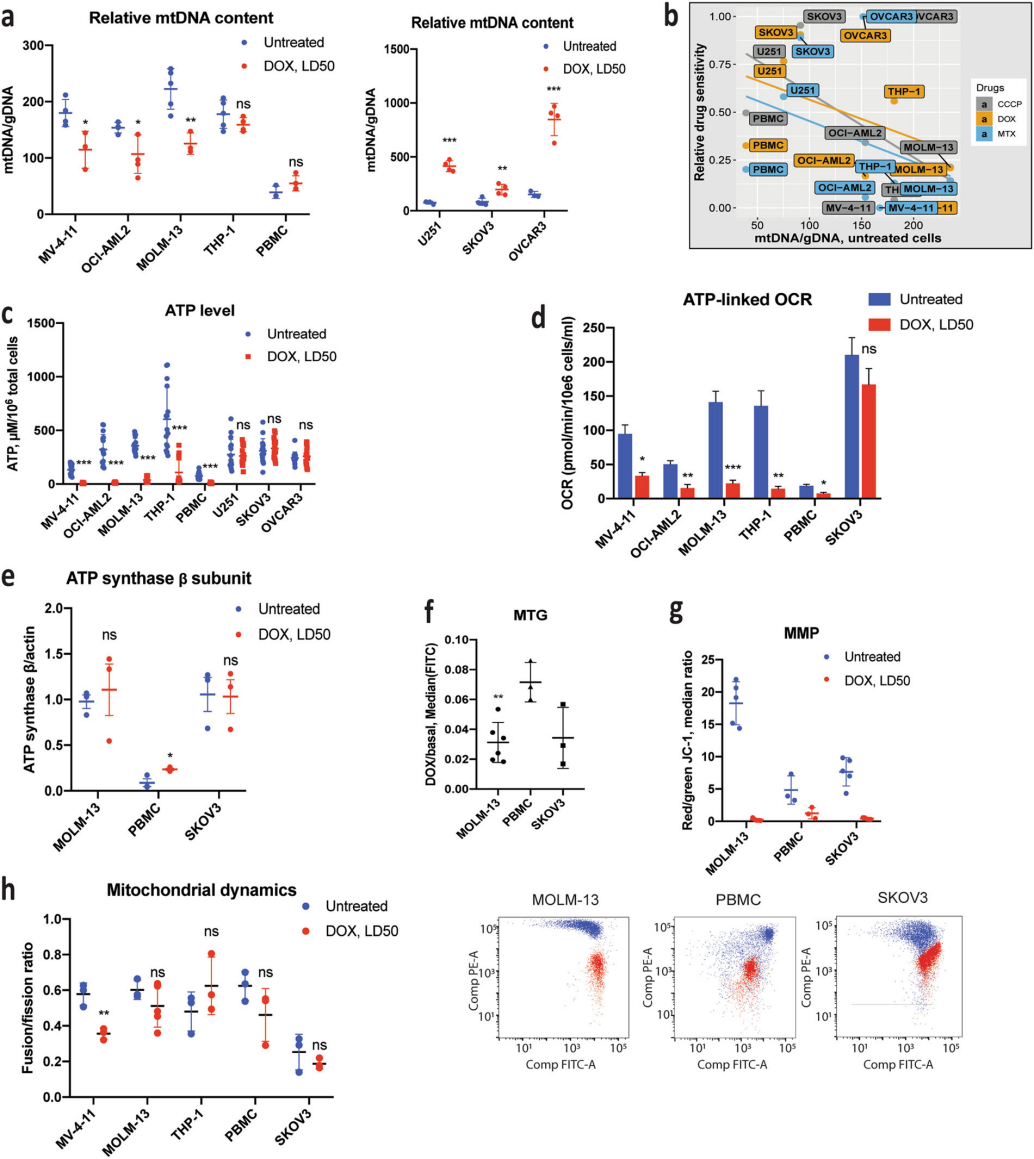
AML-derived cells are more sensitive to mitocan treatment. **a** Relative mtDNA content before and after 24 h exposure to doxorubicin treatment in AML cell lines (left) or in solid tumor cell lines (right). **b** Correlation between basal mtDNA content and LD50 of mitocans. Normalization of LD50 values was performed using min-max approach, linear functions were adjusted for the data using lm() method in R. Basal ATP level **(c)** and ATP-linked respiration **(d)** before and after exposure to doxorubicin for 24 h (**c**) or 4 h (**d**). **e** ATP synthase β subunit protein level before and after 24 h exposure to doxorubicin. β-actin was used as a loading control. **f** Changes in mitochondrial mass after 24 h doxorubicin treatment. Comparison of MOLM-13 vs. normal PBMCs is shown. **g** Mitochondrial membrane potential before and after 4 h doxorubicin treatment, as defined by JC-1 staining. Representative plots for red/green fluorescence (PE vs. FITC) after standard compensation for MOLM-13, healthy PBMCs, and SKOV3 cells are shown below for the untreated (blue) and doxorubicin-treated (red) cells. **h** Expression ratios of mitochondrial fusion/fission genes before and after 8 h doxorubicin treatment. The results of 3–6 independent experiments are presented as mean ± SD (in **a**, **c**, **f**, **g**, **h**) or mean ± SEM (in **d**, **e**). All technical replicates are shown in **c**. For statistical significance, either Student’s *t*-test with independent samples (**a**, **c**, **e**–**h**) or ANOVA with subsequent Dunn’s test (**d**) was used. ***p < 0.001; **p < 0.01; *p < 0.05; ns: *p* > 0.05

To test whether mitocan exposure has different consequences depending on cell type, we treated AML lines, healthy PBMCs, and SKOV3 cells with doxorubicin at their LD50 concentrations and assayed mitochondrial metrics. Distinct patterns of changes in mitochondrial parameters emerged. For example, leukemia cells treated with doxorubicin for 24 h displayed substantial decreases in mt/nuclear genome ratio, while solid tumor cell lines increased their mtDNA content 1.5–4-fold (Fig. 2a and Supplementary Table 6). This may reflect the increase in mtDNA copy number observed in tumor cells, which is thought to limit doxorubicin-induced apoptosis^18^. Doxorubicin showed no significant effect on mtDNA content in healthy PBMCs. Treatment with the glycolytic inhibitor 2-deoxyglucose, 2-DG, caused similar changes (Supplementary Fig. 1a). Interestingly, fitted linear regression of drug sensitivity as a function of mtDNA content was similar for three different mitocans (Fig. 2b), suggesting that cells respond to mitotoxic drugs similarly despite their different mechanism of action.

Cellular ATP levels were also examined. All leukemia cells and normal PBMCs showed depleted ATP levels after 24 h treatment with doxorubicin, while ATP levels in solid tumor cells before and after treatment were not significantly different (Fig. 2c). Similarly, a 4 h exposure to doxorubicin strongly limited oxygen consumption in leukemia cells (64–89%) and PBMCs (38%), but had no impact on ovarian cancer cells (Fig. 2d). We also measured the steady-state level of the β subunit of ATP synthase protein in MOLM-13, normal PBMCs, and SKOV3 cells after 24 h of doxorubicin treatment. Only PBMCs showed an increase in ATP-synβ after treatment; both cancer cell lines showed dramatically higher baseline levels of this protein (Fig. 2e, Supplementary Fig. 2). Sensitivity to doxorubicin showed a positive correlation with ratios of ATP (*r* = 0.929, *p* = 0.001) and mtDNA (*r* = 0.793, *p* = 0.019) (Supplementary Fig. 3).

Next, we compared changes in mitochondrial mass, mitochondrial membrane potential, Δ*Ψ*_m_, and the ratio of expression of mitochondrial fission and fusion genes induced by doxorubicin. MitoTracker Green staining (Fig. 2f) demonstrated that doxorubicin treatment for 24 h led to a loss of mitochondrial mass in all cells tested. However, this loss was significantly greater in MOLM-13 cells (32-fold) than healthy PBMCs (14-fold, *p* = 0.004). Interestingly, untreated MOLM-13 and SKOV3 cells had comparatively similar mitochondrial mass (Supplementary Fig. 4). Using JC-1 stain, which aggregates in polarized mitochondrial membranes, we assessed membrane potential in AML cells and normal PBMCs. Since JC-1 aggregation in polarized mitochondrial membranes causes a characteristic red shift in its fluorescence, the ratio of red-to-green fluorescence indicates the ratio of polarized to unhealthy mitochondria. After a 4 h treatment with doxorubicin, depolarization was much more severe in MOLM-13 cells compared to PBMC controls (64- vs. 5-fold, Fig. 2g, Supplementary Fig. 5). This drop in Δ*Ψ*_m_ is often associated with apoptosis^4,5^. It is worth noting that untreated MOLM-13 cells exhibited greater mitochondrial membrane potential than PBMCs (3.8-fold higher, *p* = 0.001) (Fig. 2g). Doxorubicin treatment also altered the ratio of expression of mitochondrial fusion/fission genes in doxorubicin-sensitive MV-4-11 cells, but not in more resistant cells (Fig. 2h, Supplementary Table 7).

### Mitochondrial toxins show synergetic effects in AML cell lines and primary AML samples

**Fig. 3:**
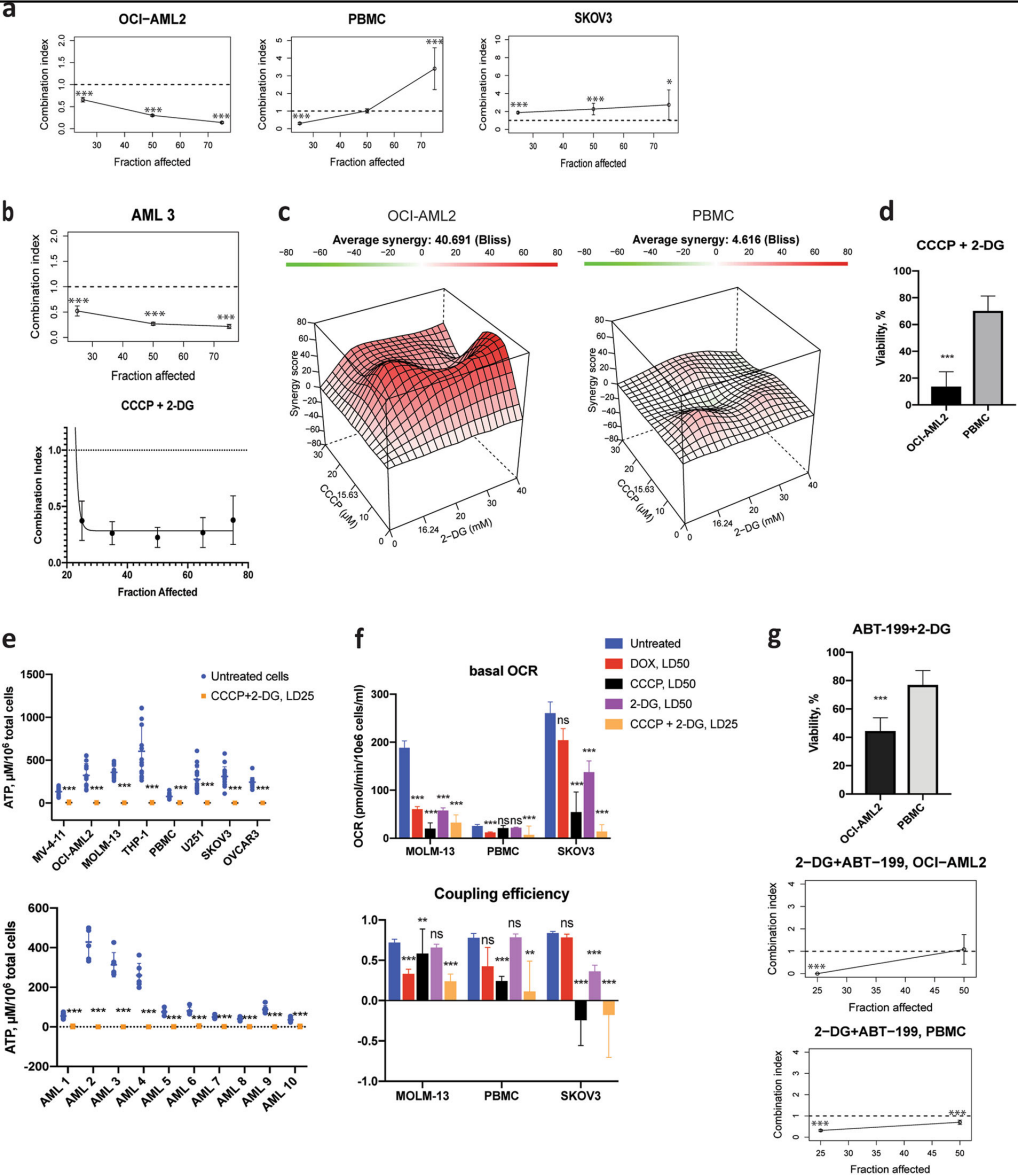
Combination therapy exhibits synergy in AML cells. FACI (combination index as a function of fraction affected) plots for combination treatment with CCCP and 2-DG in OCI-AML2, healthy PBMCs, and SKOV3 cells **(a)** or primary AML cells for one (upper) or multiple (*n* = 21, lower) patients **(b)**. Non-linear regression was used to generate a function for combined FACI data for primary AML samples. **c** Drug combination landscapes for OCI-AML2 cells and normal PBMCs built using the Bioconductor package ‘synergyfinder’^17^. **d** Viability of OCI-AML2 cells and normal PBMCs after treatment with CCCP/2-DG at LD25 concentrations corresponding to OCI-AML2 cell line. **e** ATP level before and after treatment with CCCP and 2-DG in AML cell lines, healthy PBMCs, and solid tumor cell lines (upper) or primary AML samples (lower). **f** Respiration (upper) and coupling efficiency (lower) before and after treatment with CCCP, 2-DG, or both. **g** Viability of OCI-AML2 and normal PBMC cells after treatment with ABT-199/2-DG. All graphs show results from at least three independent experiments (except **b**, where primary AML cells were limited by patient samples). FACI plots reflecting combination indices with 95%-confidence intervals were built using the Bioconductor package ‘drc’^16^, * significant difference from combination index = 1 (additivity). Elsewhere, results are presented as mean ± SD (**d**, **e**, **g**) or mean ± SEM (**f**). Statistical significance was tested using Student’s *t*-test with independent samples (**d**, **e**, **g**) or by ANOVA with subsequent Dunn’s test (**f**). ***p < 0.001; **p < 0.01; *p < 0.05; ns: *p* > 0.05

Several drug combinations based on mitotoxic drugs (such as cytarabine and mitoxantrone) have demonstrated effectiveness in AML treatment^19,20^. Therefore, we tested whether the mitochondrial uncoupler CCCP and the glycolytic inhibitor 2-DG would be effective at triggering cancer cell death. First, the relative potency of these drugs was determined for each AML cell line and patient sample (Supplementary Table 8). Trypan Blue exclusion assays were then performed using a single fixed-ratio (1:1) mixture ray design^16^. Next, combination indices based on mixtures at LD25, LD50, or LD75 were calculated. CCCP and 2-DG exhibited significant synergy (combination index < 1, *p* < 0.001) in three of four AML cell lines (MV-4-11, MOLM-13, and OCI-AML2) and in all 21 primary AML samples, but not in THP-1 or in solid tumor cell lines (Fig. 3a, b, Supplementary Figs. 6, 7 and Table 2). Interestingly, the combination was synergistic in normal PBMCs at LD25 of the mixture, but synergy disappeared at higher dosages (Fig. 3a, middle graph). Importantly, patient pretreatment did not significantly influence the potential for synergy for treatment with CCCP and 2-DG in primary AML samples (Supplementary Table 9). This was true regardless of adjustment for age, gender, source of primary cells (peripheral blood / bone marrow), or percentage of blast cells.

**Table 2:** Combination indices of the mixture of CCCP and 2-DG

Cell line/patient AML sample	Combination index (95% CI) based on LD50 of the mixture	*p*-value*	Mean combination index based on LD25, 50, 75 of the mixture
Cell lines			
MV-4-11	0.490 (0.464–0.515)	**<** **0.001**	0.529
OCI-AML2	0.302 (0.282–0.323)	**<** **0.001**	0.367
MOLM-13	0.468 (0.412–0.525)	**<** **0.001**	0.529
THP-1	1.081 (0.945–1.218)	0.244	1.106
PBMC	1.011 (0.893–1.129)	0.853	1.570
U251	1.202 (1.130–1.274)	<0.001	1.213
SKOV3	2.262 (1.621–2.903)	<0.001	2.291
OVCAR3	0.317 (0.248–0.385)	**<** **0.001**	1.146
Primary AML samples			
AML 1	0.200 (0.162–0.238)	**<** **0.001**	0.422
AML 2	0.047 (0.020–0.075)	**<** **0.001**	0.125
AML 3	0.268 (0.237–0.298)	**<** **0.001**	0.335
AML 4	0.169 (0.146–0.193)	**<** **0.001**	0.228
AML 5	0.025 (1.2 × 10^-2^–0.038)	**<** **0.001**	0.030
AML 6	0.290 (0.230–0.350)	**<** **0.001**	0.526
AML 7	0.281 (0.144–0.417)	**<** **0.001**	0.295
AML 8	0.630 (0.522–0.738)	**<** **0.001**	0.664
AML 9	0.0009 (−0.0013 to 0.0003)	**<** **0.001**	7.98 × 10^−6^
AML 10	0.034 (0.026–0.043)	**<** **0.001**	0.042
AML 11	0.154 (0.062–0.245)	**<** **0.001**	0.279
AML 12	0.295 (0.240–0.349)	**<** **0.001**	0.312
AML 13	0.161 (0.077–0.245)	**<** **0.001**	0.254
AML 14	1.3 × 10^-3^ (−0.007 to 0.009)	**<** **0.001**	0.065
AML 15	0.653 (0.363–0.943)	**0.019**	0.811
AML 16	0.521 (0.338–0.704)	**<** **0.001**	0.633
AML 17	0.263 (0.172–0.354)	**<** **0.001**	0.292
AML 18	0.243 (0.199–0.287)	**<** **0.001**	0.645
AML 19	0.404 (0.310–0.497)	**<** **0.001**	0.559
AML 20	0.077 (0.058–0.097)	**<** **0.001**	0.388
AML 21	0.003 (−0.004 to 0.010)	**<** **0.001**	0.006

Cases with *p* < 0.05 and combination index < 1 (synergy based on LD50) are highlighted in bold

^*^p-value for testing combination index based on LD50 of the mixture = 1

To optimize synergy, a set of multiple-ray treatments with assessment of cell survival was performed for CCCP and 2-DG in OCI-AML2 cells and normal PBMCs, and drug combination landscapes were determined (Fig. 3c). OCI-AML2 cells exhibited strong synergy, while PBMCs exhibited areas of additivity and antagonism, suggesting a markedly lower drug interaction. A concentration of LD25 was effective for selectively killing OCI-AML2 cells compared with PBMCs (Fig. 3d). Based on our earlier results, we measured ATP concentration after treatment with the combination of CCCP and 2-DG. Unlike doxorubicin, CCCP and 2-DG depleted ATP in every cell line studied, including primary AML samples (Fig. 3e). The combined treatment also reduced mitochondrial respiration and coupling efficiency in each of the cell lines (Fig. 3f).

To test this phenomenon further, we examined lonidamine, which compromises glycolysis (especially in neoplastic cells) and interferes with mitochondrial Complex II. Like CCCP and 2-DG, lonidamine was more toxic to AML cells than normal PBMCs or cell lines derived from solid tumors (Supplementary Fig. 1c). To investigate whether the synergetic effect of CCCP and 2-DG against AML is unique, we tested other mitocans with 2-DG. Combination of a DNA-targeted drug cytarabine (ara-C) and 2-DG also showed synergistic effect against OCI-AML2 cells at LD25 and LD50 of the mixture (Supplementary Fig. 8). It should be noted that treatment was not particularly effective against primary AML samples, even those that were not resistant to ara-C (LD25 < 400 µM, *n* = 5). Of five primary AML samples, only one (AML 20) showed significant synergy for ara-C/2-DG. Interestingly, this sample was from a patient who had been pretreated with cytarabine (Supplementary Table 3). Another mitocan, ABT-199, which targets the apoptotic inhbitor BCL-2, has shown activity in hematological malignancies, both alone and with other drugs^21,22^. ABT-199, when combined with 2-DG, showed synergy and some level of specificity to AML cells (Fig. 3g). In contrast, the combination of ABT-199 and CCCP was not selective against OCI-AML2 cells (Supplementary Fig. 9).

### Mitochondria in AML cells have high proton leak that underlies low coupling efficiency

**Fig. 4:**
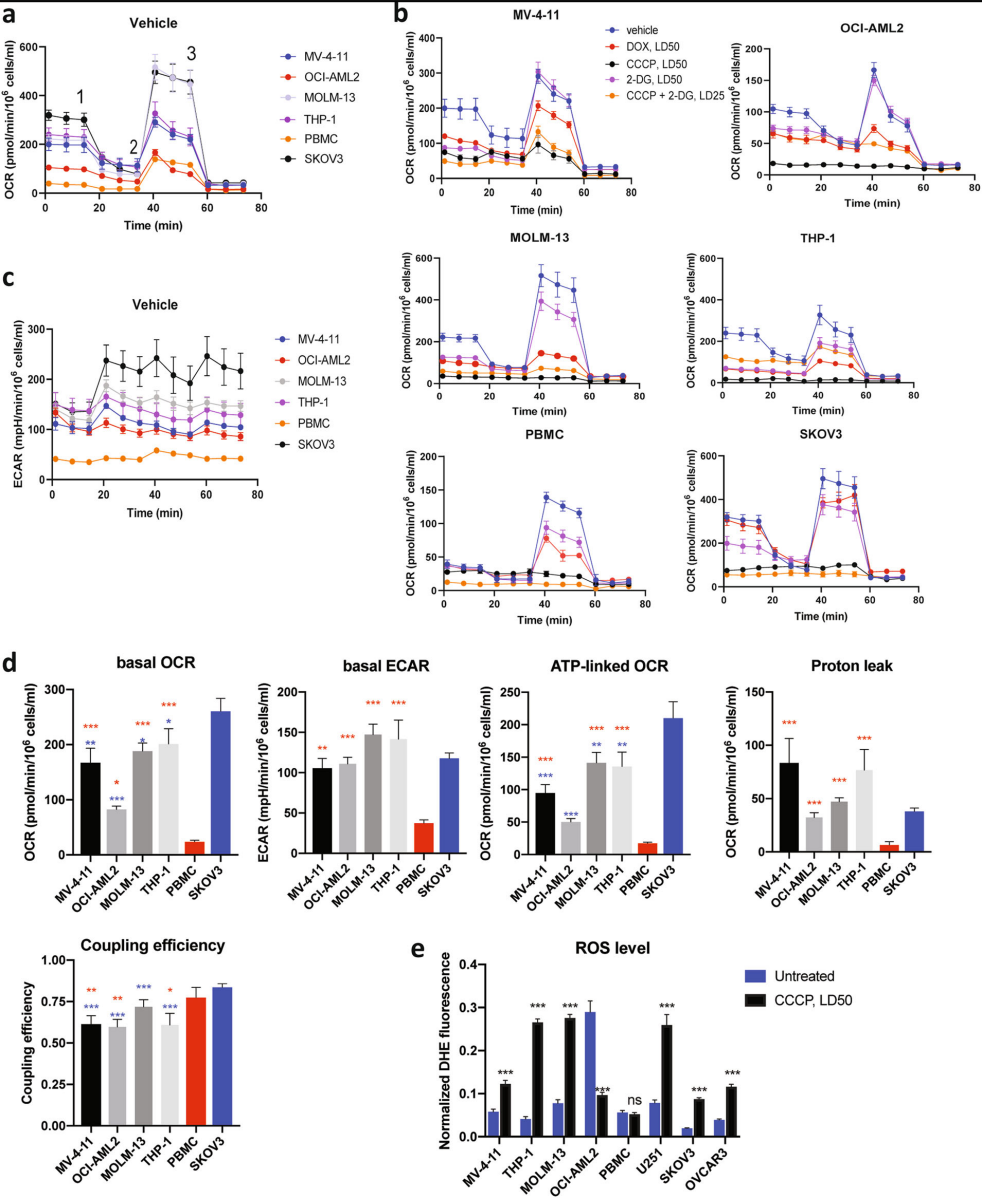
Bioenergetic profiling of AML cell lines, healthy PBMCs, and solid tumor cells. **a** Oxygen consumption rate (OCR) measured in untreated cells using a Seahorse flux analyzer. Oligomycin (1), FCCP (2), and antimycin A (3) were added at the times indicated. **b** OCR measured in cells either untreated (blue) or treated with doxorubicin (red), CCCP (black), 2-DG (purple), or CCCP and 2-DG (orange). **c** ECAR in untreated AML, PBMCs, and SKOV3 cells. **d** Basal OCR, ECAR, ATP-linked OCR, proton leak, and coupling efficiency for AML cell lines, PBMCs, and a mitocan-resistant solid tumor cell line. (red—AML vs. PBMCs, blue—AML vs. SKOV3). **e** Intracellular ROS measured by dihydroethidium (DHE, 2.5 μg/ml) after 24 h treatment with CCCP. Mean fluorescence intensities (MFI) of DHE and Hoechst 33342 were quantified using ZEN software and presented as MFI ratios DHE/Hoechst 33342. All graphs show results as mean ± SEM from 3–6 independent experiments. Statistical testing was performed by Student's *t*-test with independent samples (**e**) or ANOVA with subsequent Dunn’s or Fisher’s LSD test (**d**). ***p < 0.001; **p < 0.01; *p < 0.05; ns: *p* > 0.05

To more thoroughly test the differences in mitochondrial bioenergetics amongst our test cells (AML cell lines, healthy PBMCs, and mitocan-resistant solid tumor cells), we used a Seahorse flux analyzer to measure mitochondrial respiration in cells treated with doxorubicin, CCCP, or 2-DG (at LD50 each), or a CCCP/2-DG cocktail at LD25 (Fig. 4a–c). Each untreated AML cell line showed significantly higher basal ATP and ATP-linked respiration (*p* < 0.01), ECAR (*p* < 0.004), and proton leak (*p* < 0.008), defined as the ratio of ATP-linked respiration to basal respiration, than PBMCs (Fig. 4d). Three out of four AML cell lines had lower spare capacity (*p* < 0.04) when compared with mitocan-resistant SKOV3 cells (Supplementary Fig. 10). Intriguingly, we observed strong correlations between combination index for CCCP and 2-DG and either the baseline coupling efficiency (*r* = 0.821, *p* = 0.045) or the LD25 for either CCCP (*r* = 0.893, *p* = 0.017) or 2-DG (*r* *=* 0.869, *p* = 0.025) (Supplementary Table 10). This suggests that simple mitochondrial parameters, such as coupling efficiency, may predict drug sensitivity.

Several treatments investigated, including doxorubicin, CCCP alone, or CCCP with 2-DG had strong effects on mitochondrial respiration in hematological cells (Fig. 4b, Supplementary Table 11). In contrast, SKOV3 cells seemed to be mostly affected by the combination of CCCP and 2-DG (Supplementary Fig. 11).

We hypothesized that these effects may be related to the production of reactive oxygen species (ROS). ROS are frequently generated during mitochondrial respiration, and increases in the former are often tied to increases in the latter, unless respiration is uncoupled, which limits ROS production^23,24^. Therefore, we measured ROS levels in either untreated or CCCP-treated cells. Contrary to our expectations, treatment with CCCP significantly increased ROS in most AML cell lines, but not in PBMCs (Fig. 4e). The exception, OCI-AML2, showed a strong reduction of ROS. However, this cell line has high baseline ROS, suggesting an underlying difference in mitochondrial biology. Baseline ROS level in all AML cells was higher than that of SKOV3 cells, again indicative of significant biological differences between these cancer types (Supplementary Fig. 12). This is consistent with our other data indicating that the mitochondria in AML cells have high proton leak and low coupling efficiency that correlates with their enhanced sensitivity to the mitochondrial uncoupler CCCP, alone or in combination with other drugs.

### Mitocan treatment results in activation of multiple cell death pathways

**Fig. 5:**
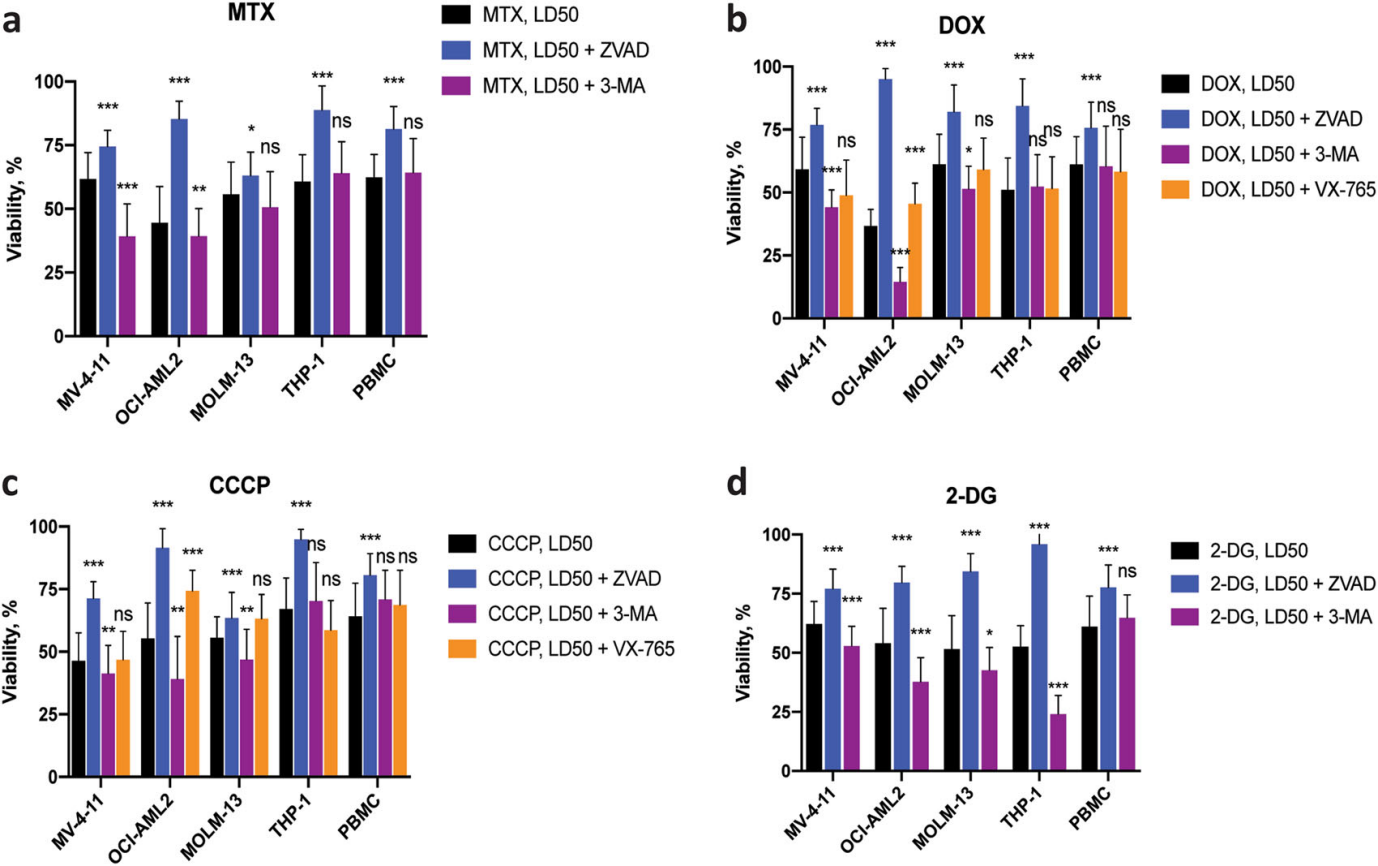
Mitocan treatment activates multiple cell death pathways. Survival rates of AML cells and healthy PBMCs treated with mitoxantrone **(a)**, doxorubicin **(b)**, CCCP **(c)**, or 2-DG **(d)** at LD50 doses. For each drug, cells were also tested with or without the following cell death pathway inhibitors: z-VAD-fmk (40 μM, pan-caspase inhibitor), 3-methyladenine (5 mM, autophagy inhibitor), or VX-765 (10 μM, caspase-1 inhibitor). Results show the mean ± SD from at least three replicates. Statistical testing was performed by Student’s *t*-test with independent samples. ***p < 0.001; **p < 0.01; *p < 0.05; ns: *p* > 0.05

Mitochondrial dysfunction is associated with the activation of a variety of cell death pathways. To determine which of these may be responsible for the death of AML cells after mitocan exposure, we assayed cell viability after treatment with MTX, DOX, and CCCP in the presence of caspase inhibitors (z-VAD-fmk for all caspases and VX-765, for caspase-1) or an autophagic inhibitor (3-methyladenine, 3-MA)^25–27^. For all cells tested, z-VAD-fmk improved viability (56.5% on average for AML cells and 21% for normal PBMCs, Fig. 5a–d). Other treatments exhibited greater variation in effect. For example, VX-765, reported to specifically inhibit caspase-1, decreased cell death only for the OCI-AML2 cells (Fig. 5a, b). In contrast, 3-MA did not change viability in THP-1 and normal PBMCs, but occasionally augmented the impact of mitocans or 2-DG. For instance, doxorubicin at the LD50 dose was significantly more detrimental for OCI-AML2 cells when autophagy was inhibited by 3-MA, and significantly less detrimental when cells were also treated with both caspase inhibitors. This outcome suggests that AML cells utilize autophagy to mitigate the mitochondrial damage occurring in these cells, consistent with previous reports^28,29^.

## Discussion

In this study, we show that leukemia cells are significantly more sensitive to mitocans than healthy PBMCs and solid tumor cell lines. AML cells possess significantly higher mitochondrial mass and mtDNA content, suggesting that they have upregulated mitochondrial activity, likely due to compensation for higher energy demands. Mitochondria in AML cells exhibit increased respiratory activity, while simultaneously having lower coupling efficiency (associated with increased proton leak) and lower spare reserve capacity. These findings are in line with previously reported studies^30–32^ and likely explain the increased sensitivity AML cells show to mitochondria-targeted drugs. In particular, we demonstrate, for the first time, that combining a mitochondrial uncoupler like CCCP and a glycolytic inhibitor, such as 2-DG, shows significant synergistic effect in AML cells, while largely leaving healthy PBMCs viable.

Doxorubicin, a well-known chemotherapeutic agent with mitochondrial toxicity^33^, has been shown to play differing roles, both inducing mitophagy that can promote chemotherapeutic resistance and damaging mtDNA which can cause cancer cell death; likely these differences depend on dose, duration, and cancer type^34–36^. Doxorubicin had pleiotropic effects on AML-derived cells, including decreasing ATP, reducing mtDNA content, decreasing mitochondrial respiration and coupling efficiency. In contrast, none of these parameters showed substantial changes in cell lines derived from solid tumors. Unfortunately, doxorubicin (and other anthracyclines) exhibit profound cardiotoxicity, which is likely at least partly a consequence of its plethora of intracellular effects.

While a cocktail of CCCP and 2-DG inhibited mitochondrial function and depleted ATP in all cell types examined, the effects were much more pronounced for hematological malignancies, when >80% of cancer cells were killed, while approximately three-quarters of normal PBMCs remained viable. We observed that AML-derived cell lines showed higher levels of glycolytic activity than the solid cancer cell lines, like SKOV3. This is probably due to the increased proton leak and mitochondrial uncoupling that were observed in AML-derived cell lines, which limit mitochondrial contribution to cellular energy production.

There are several possible explanations for the increased sensitivity of AML-derived cells to the combination of CCCP and 2-DG. First, these cells appear to be somewhat more sensitive to 2-DG than solid tumor cell lines. High doses of 2-DG have been associated with inhibition of CCCP-induced mitophagy^37^, which may promote survival. Second, even under normoxic conditions, high proton leak can promote Warburg-like metabolism^32^. With rare exceptions, ‘healthy’ mitochondria display limited proton leak^38^. Healthy mitochondria can also self-regulate, as increased ROS can induce proton conductance which can, in turn, suppress ROS production^39–41^. This may also explain the relatively low cytotoxicity of the CCCP/2-DG cocktail on normal cells at doses that kill AML cells. This understanding is also consistent with our data on SKOV3 cells. Bioenergetic profiling suggests that they primarily use OXPHOS rather than glycolysis^42^, which may explain their resistance to 2-DG. The high spare respiratory capacity of SKOV3 cells may also help them survive the low oxygen tension frequently present in solid tumor microenvironments^43^.

To the best of our knowledge, the synergistic effect of CCCP and 2-DG in cancer cells has not been reported previously, although 2-DG has been shown to exhibit synergistic effects with metformin, oligomycin, and antimycin^44,45^. Mitochondrial uncoupling by BCL-2 inhibitors BH3I-2′ and HA14-1 was synergistic with TRAIL-induced apoptosis in leukemia cells^46^. ABT-199 (venetoclax), a mitocan, and azacitidine (a nucleotide analog) have been proved to be highly active in previously untreated AML patients, eradicating leukemia stem cells^20^. Also, CPI-613, a novel inhibitor of mitochondrial metabolism via pyruvate dehydrogenase, was effective in patients with refractory AML when combined with cytarabine and mitoxantrone^19^. Furthermore, hexokinase II in leukemia cells is associated with mitochondria to attain direct access to ATP for glucose phosphorylation^32,47,48^, representing another metabolic adaptation possibly contributing to favorable response to CCCP/2-DG in leukemia cells. Our results contribute to recent findings regarding the promise of mitocans and their combinations in chemotherapy, especially in hematological malignancies. They also provide a mechanistic underpinning for their activity, which may be particularly important to consider for older patients or those with high-risk cytogenetics.

## Supplementary information

Supplemental Figures

Supplemental Tables
