# Supplementary material for: A mechanism for increased sensitivity of acute myeloid leukemia to mitotoxic drugs
Source: Cell Death Dis. 2019 Aug 13;10(8):617. doi: 10.1038/s41419-019-1851-3 (PMC6692368; doi:10.1038/s41419-019-1851-3)
Supplement: Supplementary file 1 — Supplemental Figures [file 41419_2019_1851_MOESM1_ESM.pdf]

Supplementary Figures

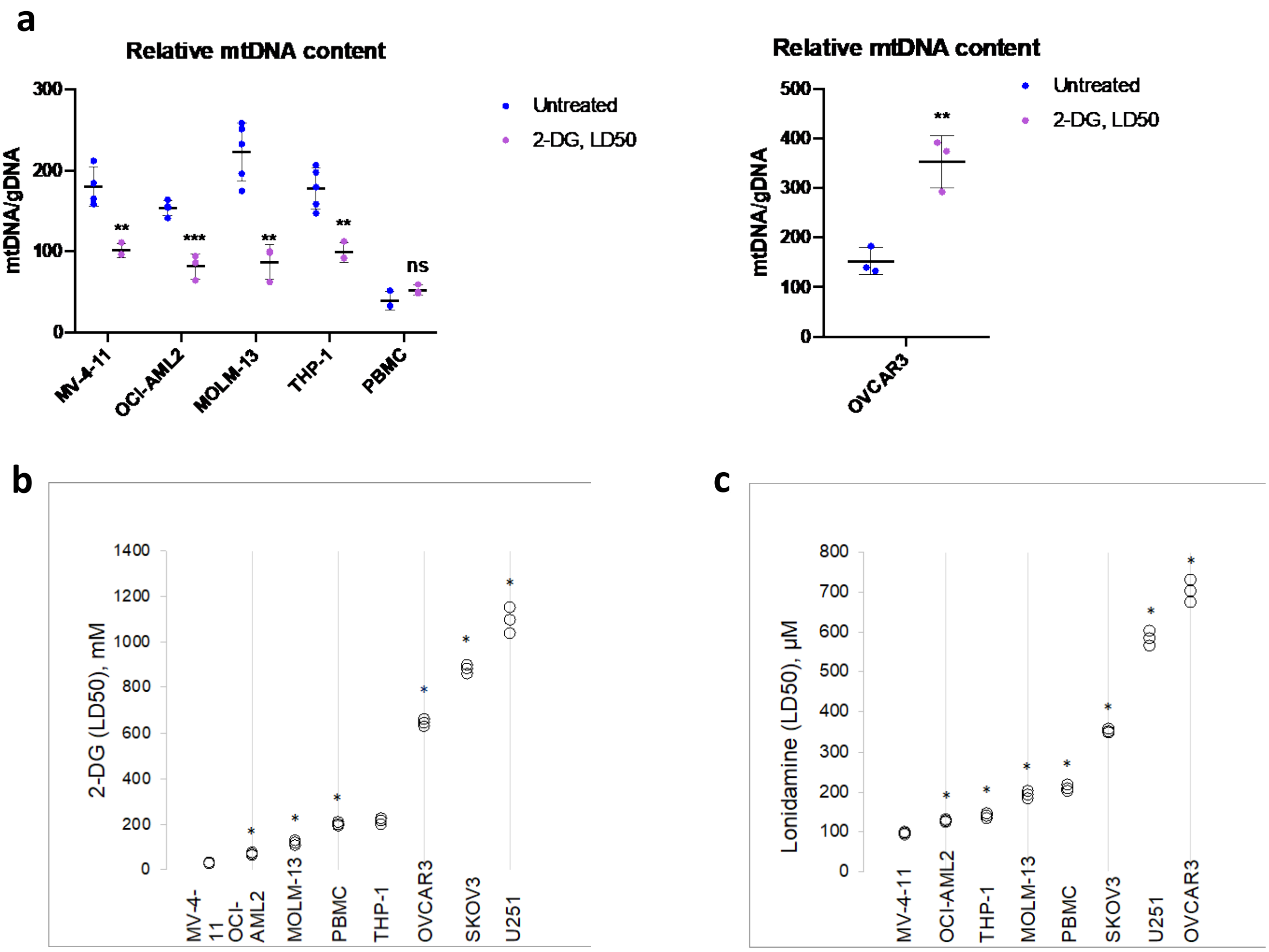

Figure S1. AML cells display higher sensitivity to glycolytic inhibition. (a) Effect of the glycolytic inhibitor 2-DG (LD50, 24 h) on mtDNA content in AML cells (left) and OVCAR3 solid tumor cells (right). SKOV3 and U251 were not analyzed due to their high resistance to 2-DG (LD50 > 880 mM). The results of 3 independent replicates are presented as mean ± SD. Significance was assessed using Student's *t*-test with independent samples. ***: $p < 0.001$; **: $p < 0.01$; ns: $p > 0.05$. **(b, c)** Sensitivity of cell lines to the glycolytic inhibitors 2-DG (**b**) and lonidamine (**c**) are shown, including LD50 and 95%-confidence intervals. Comparisons of LD50 were performed by ratio test (1). * denotes a significant difference from the cell line closest in sensitivity.

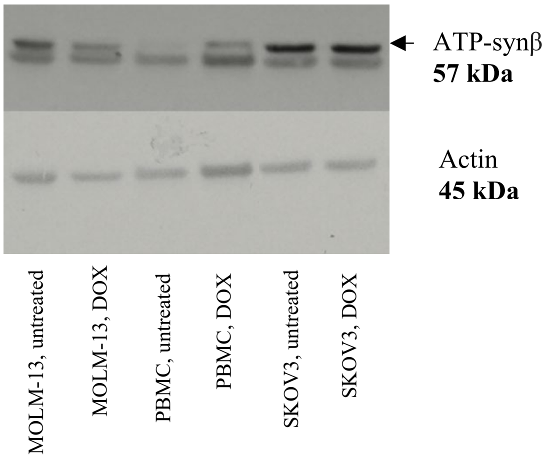

Figure S2. Increased expression of ATP synthase in cancer cells. Western blots for the amount of ATP-synβ (arrowhead, upper panel) protein in cancer cells and PBMCs with and without doxorubicin treatment. Actin (lower panel) served as a loading and normalization control.

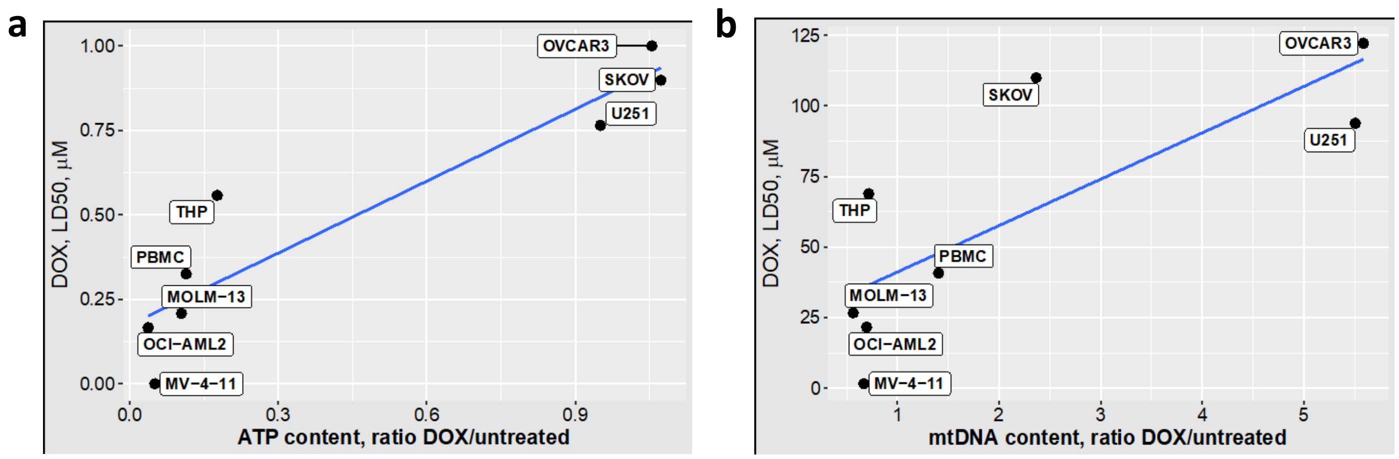

Figure S3. Sensitivity to doxorubicin correlates with ATP and mtDNA content. Correlation between sensitivity to DOX (LD50) and **(a)** the ratio of baseline ATP levels or **(b)** the ratio of mitochondrial to nuclear DNA. Linear functions were adjusted for the data using lm() method in R.

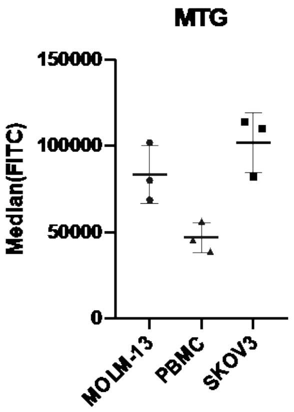

Figure S4. Mitochondrial mass does not differ between MOLM-13 and SKOV3 cells. Mitochondrial mass was inferred based on staining with MitoTrackerGreen. The results of 3 independent replicates are presented as mean ± SD. Statistical significance was calculated using Student's *t*-test with independent samples.

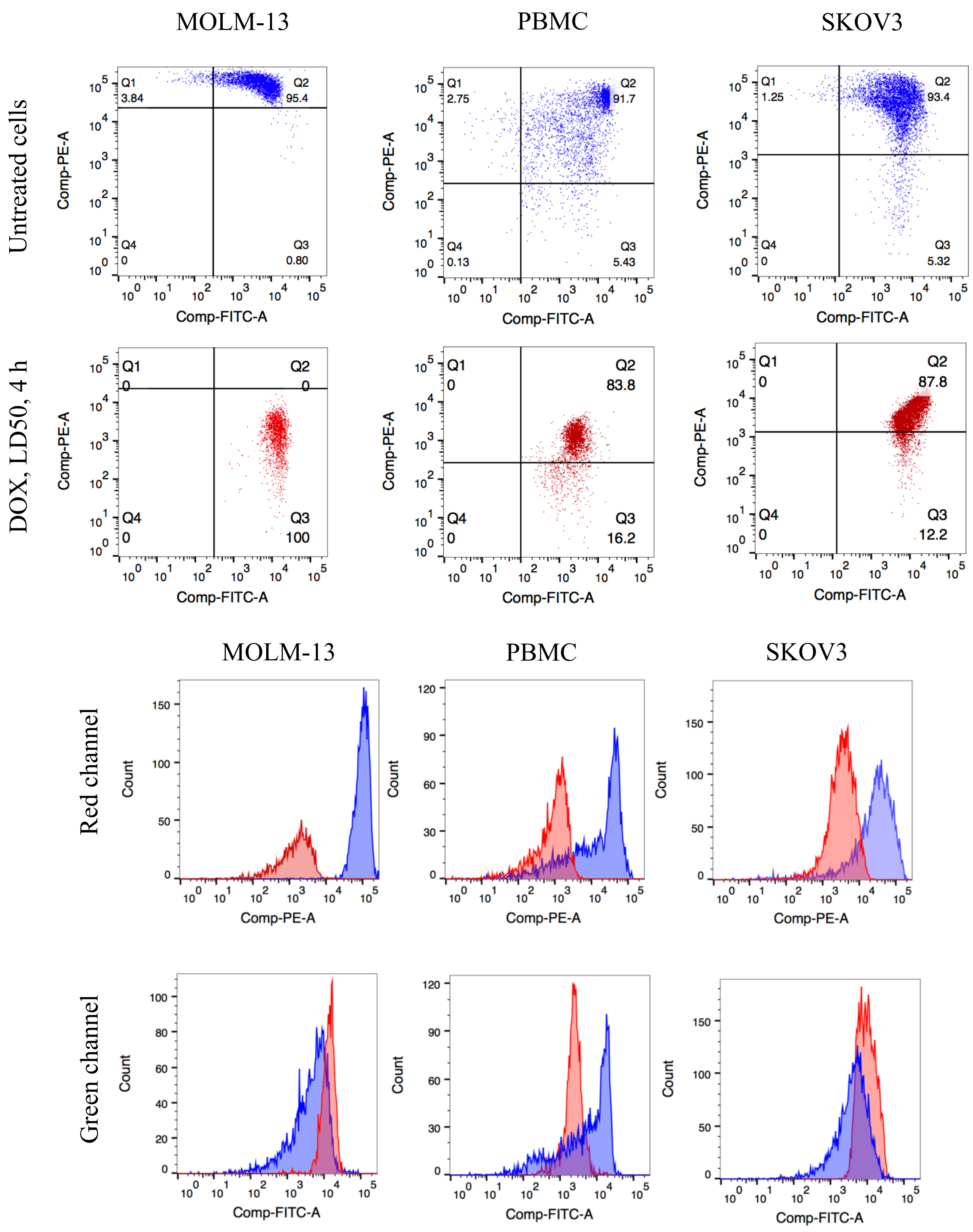

Figure S5. MOLM-13 cells exhibit the strongest change in mitochondrial membrane potential after doxorubicin treatment. Fluorescence of JC-1 in MOLM-13, PBMCs, and SKOV3 cells before and after treatment with DOX. Untreated cells are shown in blue, doxorubicin-treated cells are shown in red. One representative replicate (of three) is shown.

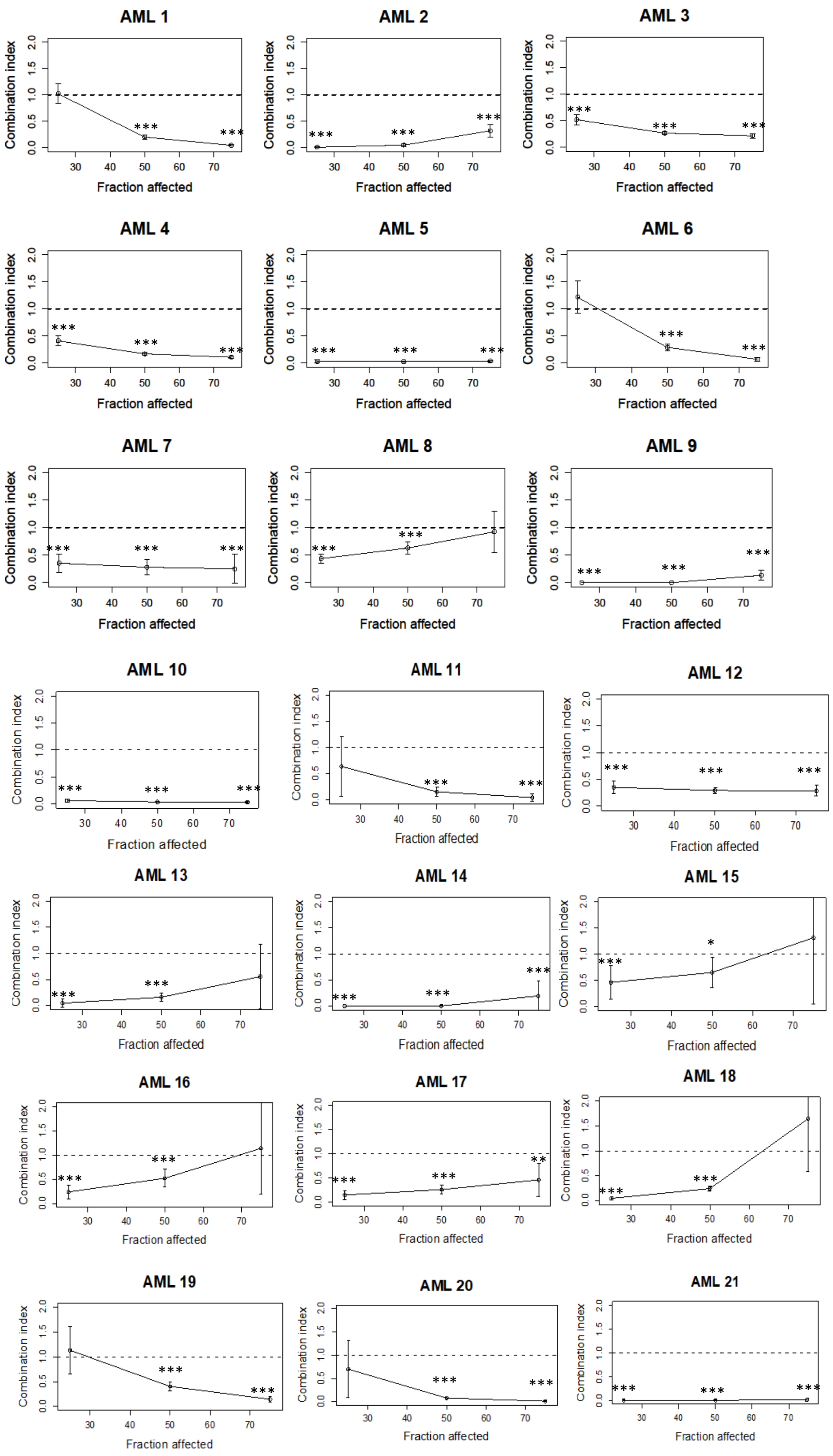

AML 1
AML 2
AML 3
AML 4
AML 5
AML 6
AML 7
AML 8
AML 9
AML 10
AML 11
AML 12
AML 13
AML 14
AML 15
AML 16
AML 17
AML 18
AML 19
AML 20
AML 21
Combination index
Fraction affected

Figure S6. Synergy of CCCP and 2-DG in primary AML cells. FACI plots for combination treatment with CCCP and 2-DG in 21 primary AML samples displaying indices with 95%-confidence intervals based on LD25, LD50, or LD75 of the mixture (results of 1-3 independent experiments shown). Analysis was done using Bioconductor package ‘drc’ (2). * $p < 0.05$, ** $p < 0.01$, *** $p < 0.001$: significant difference from combination index = 1 (additivity).

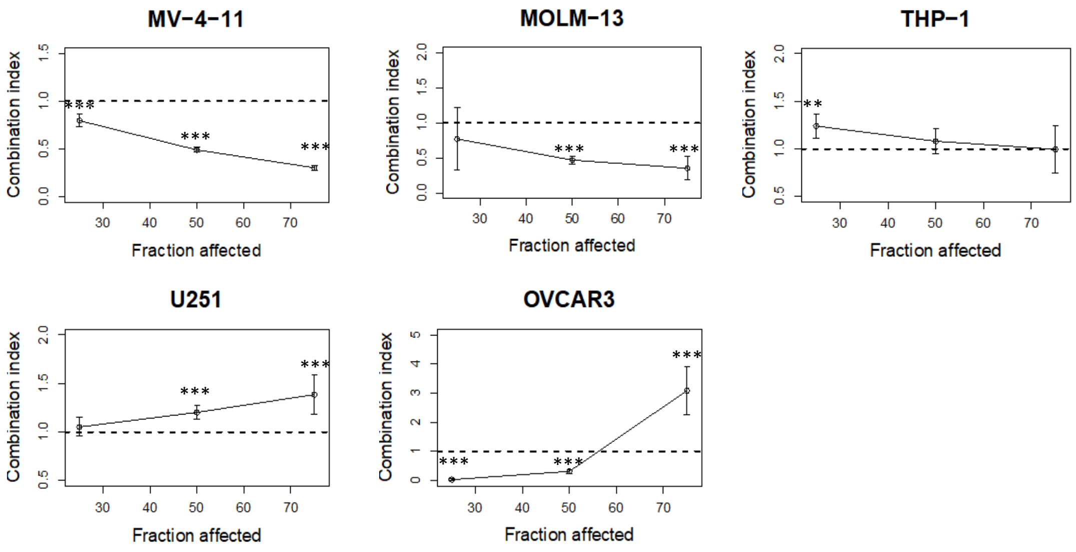

Figure S7. Combination of CCCP and 2-DG shows different interaction profile in AML and solid tumor cells. FACI plots for combination treatment with CCCP and 2-DG in AML cell lines (MV-4-11, MOLM-13, THP-1) and solid tumor cells (U251, OVCAR3). Combination indices are shown with 95%-confidence intervals, based on LD25, LD50, or LD75. Results shown are from 3 independent experiments shown. Analysis was performed as in Figure S6. ** $p < 0.01$, *** $p < 0.001$: significant difference from combination index = 1 (additivity).

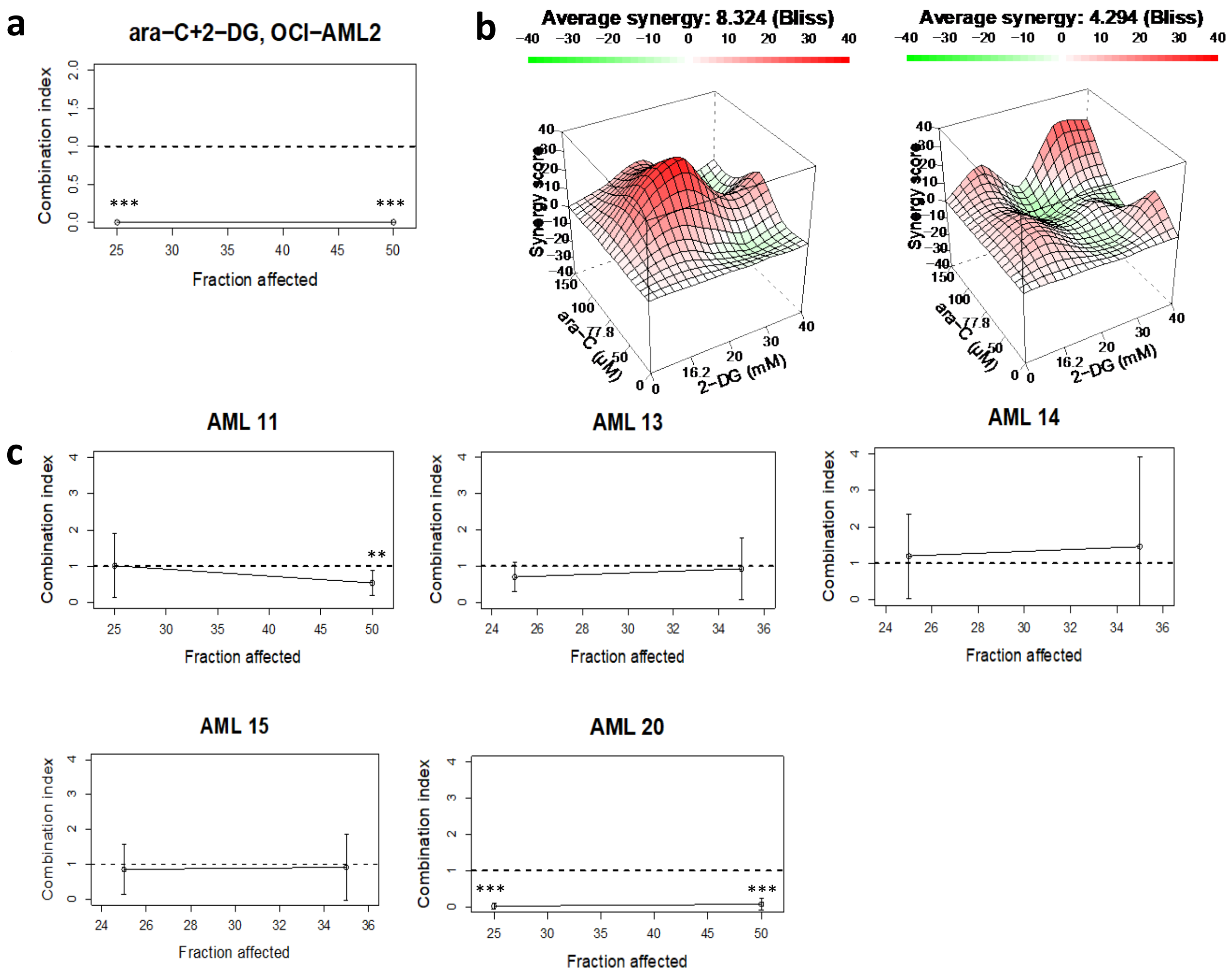

Figure S8. Combination treatment with cytarabine (ara-C) and 2-DG in OCI-AML2, PBMC, and primary AML samples. (a) FACI plot for OCI-AML2 cell line reflecting combination indexes with 95%-confidence intervals based on LD25 and LD50. **(b)** Drug combination landscapes for OCI-AML2 cells (left) and normal PBMCs (right) built using Bioconductor package 'synergyfinder' (3). **(c)** FACI plots for primary AML samples reflecting combination indexes with 95%-confidence intervals based on LD25 and LD50 (AML 11,20); LD25 and LD35 (AML 13,14,15). Only primary AML samples, not resistant to ara-C (LD25 < 400 µM, n = 5), have been analyzed. Results show 3 independent experiments (primary AML samples – 1-3 independent experiments). **a, c** Analysis was performed as in Figure S6. ** $p < 0.01$, *** $p < 0.001$: significant difference from combination index = 1 (additivity).

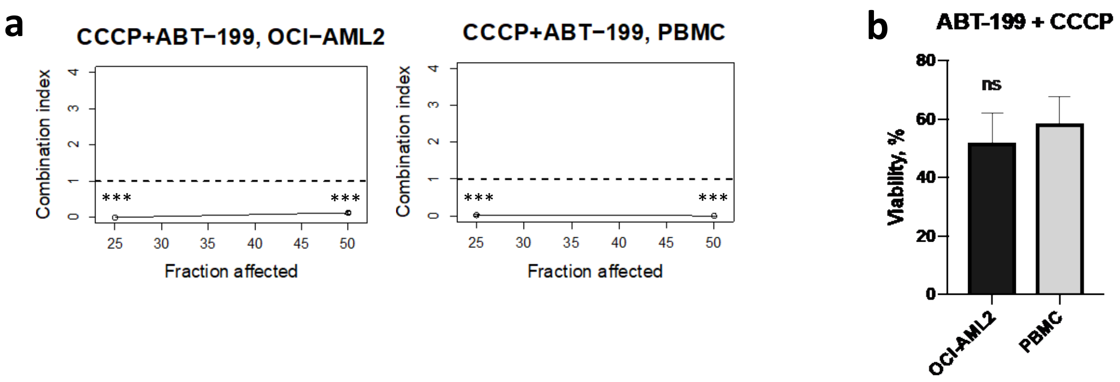

Figure S9. Combination treatment with CCCP and ABT-199 in OCI-AML2 and PBMC cells. (a) FACI plots reflecting combination indexes with 95%-confidence intervals based on LD25 and LD50. Results show 3-5 independent experiments. Analysis was performed as in Figure S6. *** $p < 0.001$: significant difference from combination index = 1 (additivity). **(b)** Survival of AML and normal PBMCs after treatment with CCCP and ABT-199. Results are shown as mean ± SD. Student's *t*-test with independent samples was used to test significance. ns: $p > 0.05$.

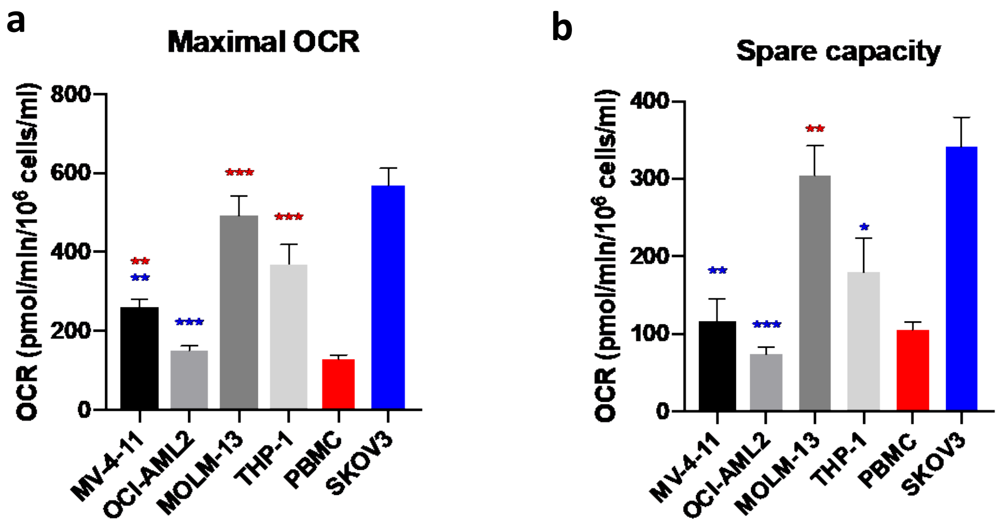

Figure S10. Maximal OCR and spare capacity in cancer and PBMC cells. Maximal respiration induced by FCCP acute treatment **(a)** and spare capacity **(b)** in untreated cells. The results are shown as mean ± SEM of 3-6 independent experiments. The groups were compared by ANOVA with subsequent Dunn's or Fisher's LSD test. * $p < 0.05$, ** $p < 0.01$, *** $p < 0.001$, ns: $p > 0.05$.

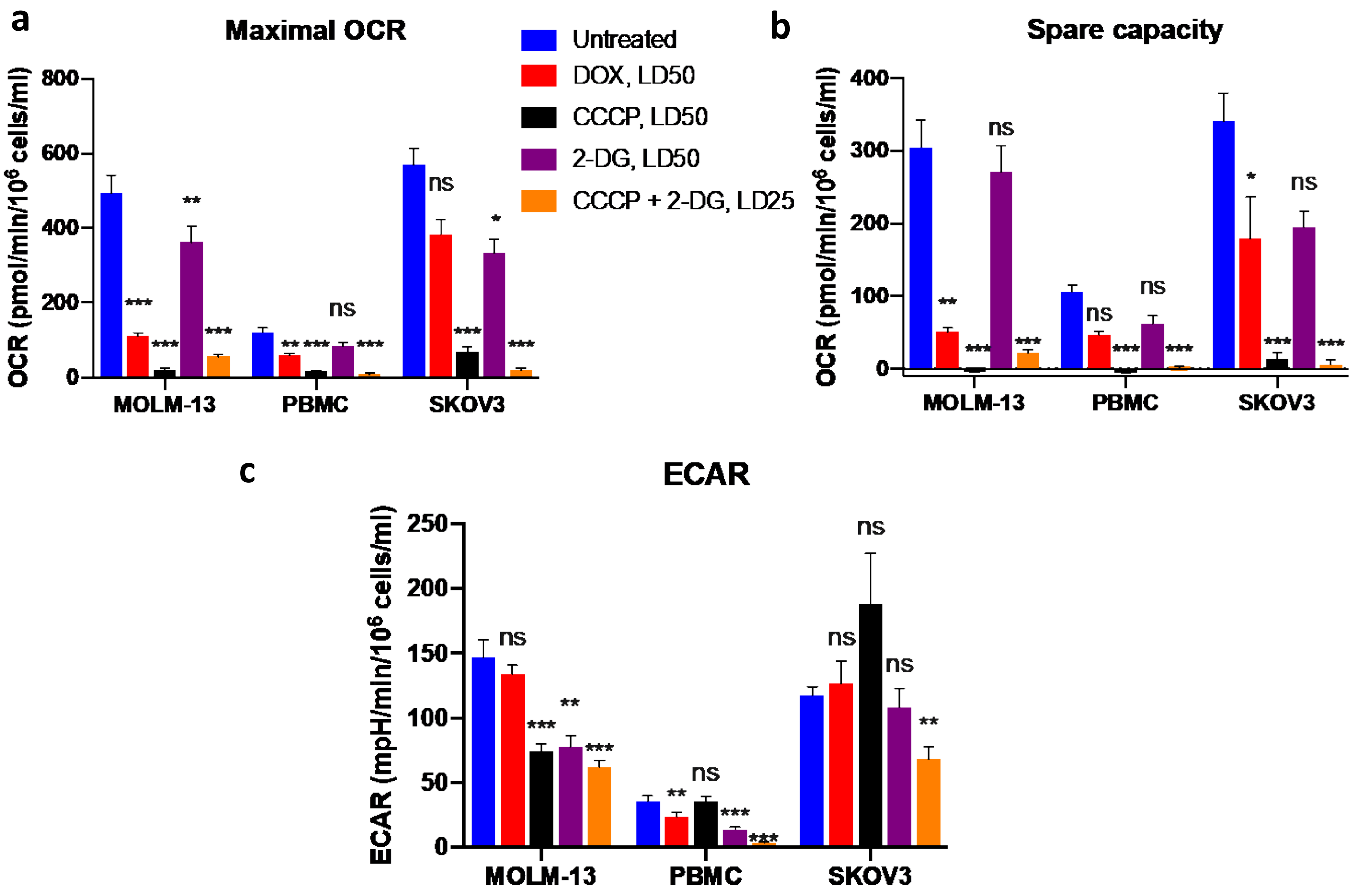

Figure S11. Mitocan treatment reduces mitochondrial function. AML, PBMC, and mitocan-resistant SKOV3 cells respond differently to treatment with mitocans (CCCP, DOX), a glycolytic inhibitor (2-DG) or their combination (CCCP+2-DG) for 4 h. Maximal respiration **(a)**, spare capacity **(b)**, and ECAR values **(c)** are shown as mean ± SEM of 3-6 independent experiments. The groups were compared by ANOVA with subsequent Dunn's or Fisher's LSD test. * $p < 0.05$, ** $p < 0.01$, *** $p < 0.001$, ns: $p > 0.05$.

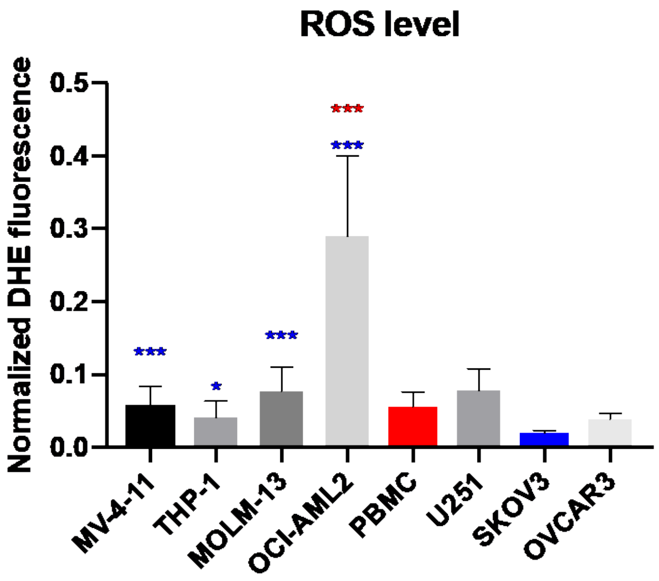

Figure S12. Basal level of ROS in a panel of cancer cells and PBMCs. ROS level in untreated cells represented by dihydroethidium (DHE) staining, normalized to nuclear (Hoechst 33342) staining. Mean fluorescence intensities (MFI) of DHE and Hoechst 33342 were quantified using ZEN software and presented as MFI ratios DHE/Hoechst 33342. ROS level is shown as mean±SD of 3 independent experiments. The groups (AML vs. PBMC) were compared by ANOVA with subsequent Dunn's test (in red – AML vs. PBMC, in blue – AML vs. SKOV3). *** $p < 0.001$; * $p < 0.05$.

References

1. Wheeler MW, Park RM, Bailer AJ. Comparing median lethal concentration values using confidence interval overlap or ratio tests. *Environ Toxicol Chem.* 2006;**25**(5):1441-4.

2. Ritz C, Streibig JC. From additivity to synergism – A modelling perspective. *Synergy.* 2014;**1**(1):22-9.

3. Ianevski A, He L, Aittokallio T, Tang J. SynergyFinder: a web application for analyzing drug combination dose-response matrix data. *Bioinformatics.* 2017;**33**(15):2413-5.
