# Supplementary material for: A mechanism for increased sensitivity of acute myeloid leukemia to mitotoxic drugs
Source: Cell Death Dis. 2019 Aug 13;10(8):617. doi: 10.1038/s41419-019-1851-3 (PMC6692368; doi:10.1038/s41419-019-1851-3)
Supplement: Supplementary file 2 — Supplemental Tables [file 41419_2019_1851_MOESM2_ESM.pdf]

Supplementary Tables

Supplementary Table S1. List of mitocans and non-mitocans used for bioinformatics analysis.

Mitocans	*Non-mitocans*
Cytarabine	Hydroxyurea
Doxorubicin	Fludarabine
Paclitaxel	BN-2629
Etoposide	E-7820
Rapamycin	Fulvestrant
Vinorelbine	Dasatinib
Temsirolimus	Pazopanib
Arsenic trioxide	Afatinib
Obatoclax	Dolastatin 10
Salinomycin	Estramustine
Staurosporine	Midostaurin
Simvastatin	Testolactone
Mitoxanthrone	Valrubicin
L-Asparaginase	Fostamatinib

Supplementary Table S2. List of cell lines included in the study.

Cell line	Cell line origin	Disease	Age	Sex	Prior treatment	Ploidy	Genetic mutations / rearrangements
MV-4-11	Peripheral blood	Acute myeloid leukemia	10	M	NA	2n=48 (hyperdiploid)	FLT3-ITD[1], t(4;11)[2] [PMID: 12529668]
OCI-AML2	Peripheral blood	Acute myeloid leukemia	65	M	NA	Hyperdiploid 48(43-49)	*DNMT3A* [PMID: 21904384]
THP-1	Peripheral blood	Acute myeloid leukemia	1	M	NA	Near-diploid (46, XY)	*MLL-AF9* fusion, deletions in *CDKN2A, PTEN* [PMID: 11066077, 19635138]
MOLM-13	Peripheral blood	Acute myeloid leukemia	20	M	NA	Hyperdiploid 51(48-52)	FLT3-ITD, CBL deltaExon8 mutant, *MLL-AF9* fusion [PMID: 12529668; 9305600]
U251	Central nervous system	Glioblastoma	75	M	NA	2n=46	*CDKN2A, C9orf53, PTEN, TP53* [PMID: 24810477; 17088437]
SKOV3	Ovary	Adeno-carcinoma	64	F	Thiotepa	Hypodiploid (42-45)	*TP53, CDKN2A, PIK3CA, APC* [PMID: 26585234; 17088437]
OVCAR3	Ovary	Adeno-carcinoma	60	F	CyPh/CsPt/Adr	Aneuploid, near-triploid 69+/-	*TP53* [PMID: 17088437]

[1] internal tandem duplication

[2] chromosomal translocation

Supplementary Table S3. Basic characteristics of the patients, enrolled in the study.

N	PB/BM[1]	age	sex	WBC	% blast	Pretreatment	Mutation status: gene mutations/genetic rearrangements	Karyotype
1.	PB	29	F	4.6	36	Decitabine, Venetoclax, Dasatinib	PTPN11, CSF3R	48,XX,-11,del(17)(p12),+r,+2mar[2]/49,idem,+8[12]/48~50,XX, t(7;11)(q22;q24),+8,del(9)(q13q22),-11,del(17)(p12),+r,+2~3mar[cp6]
2.	PB	83	M	15.6	30	ONC201, Cytarabine	TERT, CSF3R, TP53, GATA2	45,XY,t(3;8)(q25;q22),-5,del(7)(q22q34),add(8)(q24.1), del(12)(p11.2),16,+mar[18]/46~49,idem,+del(7)(q22q34)[cp2]
3.	PB	35	F	23.2	62	Decitabine	ASXL1, ASXL2, NRAS, RUNX1, FLT3 ITD, PHF6	46, XX
4.	PB	53	M	2.1	63	DCLL9718S	KMT2A, U2AF1	46,XY,add(1)(p36.1),del(7)(q22q34),del(20)(q11.2q13.3)[19]/47,idem,+del(7)(q22q34)[1]
5.	PB	41	F	13.5	92	Hydroxyurea, Crenolanib, Decitabine, Venetoclax, Gilteritinib	TET2, FLT3 ITD	49,XX,+4,t(6;9)(p22;q34),+8,+15,del(15)(q11.2q21)x2[14]/48~50,idem,+13[cp6]
6.	PB	77	M	4.9	57	Azacitidine, Cytarabine, Cladribine, Venetoclax	TET2, SRSF2, RUNX1, CBL, IL7R	46, XY
7.	PB	32	F	12.7	5.5	No treatment	FLT3 ITD, NPM1, DNMT3A	46, XX
8.	PB	57	M	37.2	15	Hydroxyurea. Cytarabine	ASXL1, NRAS, TET2, RUNX1T1	47,XY,+19[1]/47,idem,der(22)t(8;22)(q21.2;p11.2)[11]/46,XY[8]
9.	PB	79	F	5.4	6	IMGN622	ASXL, BCOR, DNMT3A	46,XX,t(2;17)(p14;q12),add(10)(q24),del(21)(q22q22)[19]/46,XX[1]
10.	BM	80	M	128	89	Hydroxyurea	FLT3 ITD, RUNX1 ISH2, PIGA, DNMT3A, ZRSR2	46, XY
11.	BM	57	M	9.3	86	No treatment	SMC1A, SRSF2, IDH1, ASXL2, FLT3 ITD	47,XY,+8[3]/46,XY,add(5)(q35)[2]/48,XY,+8,+mar[7]/47,XY,add(5)(q35),+8[6]/48,XY,add(5)(q35),+8,+mar[1]/49,XY,+8,+2mar[1]
12.	PB	64	M	3.1	67	Decitabine	TET2, TP53, EZH2, CBL	41~45,Y,der(X)t(X;1)(q24;p13),-2,add(3)(p13),del(5)(q13q35),-8,psu dic(9;10)(p22;p11.2),-15,-17,add(17)(p11.2), add(21)(p11.2),del(22)(q11.2),+2~6mar[cp19]/46,XY[1]
13	PB	58	M	38	31	No treatment	ETV6, EZH2, NRAS, SETBP1, DNMT3A	46, XY
14.	PB	70	M	8.2	48	No treatment	KDM6A, RUNX1, TP53	46~47,XY,+1,der(1;10)(q10;q10),der(1;12)(p10;q10),del(5)(q11.2),del(6) (q15q25),-14,-16,add(16)(p11.2),-17,-21,+3~6mar[cp15]/46,XY[4]
15.	BM	89	M	95.8	65	No treatment	ASXL1, CBL, CREBBP, PHF6, TET2	50,XY,+der(1;11)(q10;q10),+8,+8,+8[10]/47~51, XY,+add(1)(p13),+8,+8,+8,der(9)t(1;9)(q21;p24)[cp10]
16.	BM	58	M	38	31	No treatment	ETV6, EZH2, NRAS, SETBP1, DNMT3A	46, XY
17.	PB	64	M	9.2	23	Dasatinib, Decitabine, Venetoclax	ASXL1	46,XY,add(2)(q12),t(4;12)(q12;q24.1),ins(12;?)(p13;?)[18]/46,XY[2]

18.	PB	72	M	5.2	90	AMG 330, Tocilizumab, Hydrea	BCOR, U2AF1, DNMT3A	46, XY
19.	PB	21	M	4.8	86	Decitabine, Venetoclax,	NPM1, IDH2	46,XY,t(2;4)(p23;q21)[3]/46,XY,t(5;17)(q13;q25)[2]/46,XY[15]
20.	PB	30	M	2.3	61	Mitoxantrone, Etoposide, Cytarabine	BCOR, PTPN11	45,XX,t(3;3)(q21;q26.2),del(6)(q12),-7[16]
21.	PB	72	M	1.4	38	Hydroxyurea, Quizartinib, Decitabine	CEBPA, FLT3 ITD, KRAS, PTPN11, WT1	46, XY

[1] Source of primary AML cells: PB (peripheral blood) / BM (bone marrow).

Supplementary Table S4. Primer sequences used for qPCR studies.

<table>
<tr><td>Locus</td><td>Forward (5’-3’)</td><td>Reverse (5’-3’)</td><td>PCR protocol</td></tr>
<tr><td colspan="3">Primers used for estimation of relative mtDNA content (mtDNA/gDNA ratio)</td><td rowspan="17">I. 95 ^{0}C for 30 s;
II.
1. 95 ^{0}C for 10 s;
2. 55 ^{0}C for 45 s;
+Plate read
(40 cycles)
III. Melt Curve 65 to 95 ^{0}C, increment 0.5 ^{0}C for 5 s
+Plate read
END</td></tr>
<tr><td>GAPDH</td><td>TCTGACTTCAACAGCGACAC</td><td>CAGCCACATACCAGGAAATG</td></tr>
<tr><td>B2M</td><td>TGTAAGCAGCATCATGGAGG</td><td>CTCCACATAGTGAGGGTTATC</td></tr>
<tr><td>TBP</td><td>GAGAGTTCTGGGATTGTACC</td><td>CCATCTGAAAACAGAGCAGG</td></tr>
<tr><td>ACTB</td><td>AAAGACCTGTACGCCAACAC</td><td>GGAAAGACACCCACCTTGAT</td></tr>
<tr><td>ND1</td><td>GTATTATACCCACACCCACC</td><td>GTACAATGAGGAGTAGGAGG</td></tr>
<tr><td>ND4</td><td>CACATATGGCCTAGACTACG</td><td>GTGGGTGGTTGTGTTGATTC</td></tr>
<tr><td colspan="3">Primers used for gene expression studies</td></tr>
<tr><td>MFN1</td><td>TCTCCGCCTTTAACTTCTCG</td><td>TTATGCTAAGTCTCCGCTCC</td></tr>
<tr><td>MFN2</td><td>GCAGGACATGATAGATGGCT</td><td>ATAGTTGAGGGAGAAGCACTG</td></tr>
<tr><td>OPA1</td><td>GTCAGTCAAATGGACCCTCA</td><td>ATCTGCTGAATCCTGCTTGG</td></tr>
<tr><td>DNML1</td><td>GAACAGCGAGATTGTGAGGTT</td><td>TGCATTACTGCCTTTGGCAC</td></tr>
<tr><td>FIS1</td><td>ATCCGTAAAGGCATCGTGCT</td><td>AGAAGACGTAATCCCGCTGT</td></tr>
<tr><td>MFF</td><td>ACCTGACTGTTGTAGATGCAG</td><td>AGCACGTTCTTTGTTCTCCTC</td></tr>
<tr><td>MID49</td><td>GTGAAGCGGTTCATTGACAG</td><td>TTGAGCAGGCTCAGTTCCTT</td></tr>
<tr><td>MID51</td><td>ATGTACTTGAGTGGCAGCCT</td><td>GGGCACAATGAGTTGGATGT</td></tr>
<tr><td>ACTB</td><td>GATCAAGATCATTGCTCCTCCTG</td><td>ATACTCCTGCTTGCTGATCCA</td></tr>
</table>

Supplementary Table S5. Results of multiple linear regression: association of cancer type with the sensitivity to mitocan treatment.

Dependent variable[1]	B-coefficient	SE(B)	*p*-value
After adjustment for age, gender and prior treatment of the cell line donor			
Mitocan activity Z-scores	0.382	0.168	**0.031**
Non-mitocan activity Z-scores	0.346	0.171	0.054

[1] With cancer type as an independent predictor

$p < 0.05$ are highlighted in bold

Supplementary Table S6. Fold changes (positive – increase; negative – decrease) of mitochondrial and bioenergetic parameters after doxorubicin treatment (DOX/untreated).

Cell line	mtDNA content	MTG fluore-scence	JC-1, red/ green	ATP content	Basal OCR	ATP-linked OCR	Proton leak	Spare capacity	Coupling efficiency	ECAR	WB (ATP syn ß)	LD50 (DOX), µM
MV-4-11	**-1.49**			**-19.71**	**-2.19**	**-2.84**	-1.94	-1.10	-1.43	1.22		1.634
OCI-AML2	**-1.44**			**-26.80**	**-2.02**	**-3.26**	-1.28	**-4.26**	**-2.22**	-1.03		21.67
MOLM-13	**-1.77**	**-32.03**	**-63.96**	**-9.57**	**-3.11**	**-6.29**	-1.23	**-5.95**	**-2.16**	-1.10	1.118	26.75
THP-1	**-1.40**			**-5.66**	**-5.32**	**-9.26**	**-3.30**	**-3.80**	**-2.36**	**-1.92**		68.70
PBMC	1.40	**-13.97**	**-4.71**	**-8.76**	**-1.55**	**-1.61**	-1.41	**-2.23**	-1.20	**-1.52**	**3.772**	40.85
U251	**5.51**			-1.05								122.22
SKOV3	**2.37**	**-29.19**	**-15.34**	1.07	-1.28	-1.26	-1.03	**-1.90**	-1.07	1.09	1.002	110.82
OVCAR3	**5.59**			1.05								93.87

Cases with $p<0.05$ are in **bold**.

Supplementary Table S7. Fold change differences in expression of fusion and fission genes in DOX-treated (LD50, 8 h) cells compared to untreated cells.

Gene / Cell line	Fusion			Fission				
	MFN1	*MFN2*	*OPA1*	*DNM1L*	*FIS1*	*MFF*	*MID49*	*MID51*
MV-4-11	0.83 (0.50-1.37)*	2.13 (1.34-3.39)	1.35 (0.84-2.18)	2.06 (1.26-3.36)	3.06 (1.88-4.99)	1.99 (1.19-3.34)	5.71 (3.52-9.27)	2.86 (1.69-4.85)
MOLM-13	1.09 (0.53-2.26)	2.19 (0.76-6.32)	0.51 (0.25-1.03)	0.58 (0.24-1.38)	0.80 (0.30-2.12)	0.53 (0.28-1.01)	16.82 (4.96-56.95)	1.41 (0.42-4.71)
THP-1	5.43 (3.71-7.95)	6.95 (3.61-13.37)	0.71 (0.52-0.98)	0.36 (0.13-0.99)	1.84 (1.26-2.69)	0.66 (0.48-0.92)	32.98 (15.96-68.15)	3.14 (1.78-5.54)
PBMC	3.09 (1.11-8.63)	4.82 (1.18-19.67)	1.29 (0.47-3.59)	0.91 (0.45-1.84)	1.52 (0.74-3.10)	2.24 (1.04-4.84)	13.67 (2.79-67.09)	4.72 (1.08-20.68)
SKOV3	0.33 (0.11-0.95)	0.67 (0.51-0.86)	0.46 (0.17-1.21)	0.52 (0.18-1.51)	0.93 (0.72-1.21)	0.63 (0.35-1.12)	0.58 (0.37-0.89)	0.25 (0.21-0.30)

* Fold changes were calculated using the comparative $\Delta\Delta C_t$ method

Supplementary Table S8. Relative potency of drugs used for combination studies (single-ray design) in cell lines and primary AML samples.

Cell line/Patient sample	$LD25_{1st\ drug}$ / $LD25_{2nd\ drug}$ = Relative potency	Fixed ratio (50:50) concentrations of the 1st drug, 2nd drug
2-DG (1st drug) / CCCP (2nd drug)		
MV-4-11	7500 µM / 2.07 µM = 3623	10.7 mM, 3 µM; 7.5 mM, 2.1 µM; 3.6 mM, 1 µM
OCI-AML2	16240 µM / 15.63 µM = 1039	16.2 mM, 15.6 µM; 8.1 mM, 7.8 µM; 6.55 mM, 6.3 µM
MOLM-13	51600 µM / 6.3 µM = 8190	51.6 mM, 6.3 µM; 41 mM, 5 µM.
THP-1	74800 µM / 4.06 µM = 18424	74.8 mM, 4.1 µM; 36.8 mM, 2 µM; 18.4 mM, 1 µM
PBMC	106000 µM / 45.41 µM = 2334	106 mM, 45.41 µM; 70 mM, 30 µM; 46.7 mM, 20 µM; 23.3 mM, 10 µM
U251	616150 µM / 92.3 µM = 6676	616.15 mM, 92.3 µM; 467.3 mM, 70 µM; 333.8 mM, 50 µM
SKOV3	737000 µM / 133.4 µM = 5525	800 mM, 139 µM; 737 mM, 133.4 µM; 688.9 mM, 120 µM
OVCAR3	390748 µM / 130.3 µM = 2999	450 mM, 150 µM; 390.7 mM, 130.3 µM; 300 mM, 100 µM; 150 mM, 50 µM; 30 mM, 10 µM
AML 1	8660 µM / 12.33 µM = 702	8.66 mM, 12.33 µM; 5.77 mM, 8.22 µM; 2.87 mM, 4.11 µM
AML 2	79100 µM / 16.07 µM = 4922	79 mM, 16.07 µM; 50 mM, 10 µM; 24.5 mM, 5 µM
AML 3	80700 µM / 11.4 µM = 7079	80.7 mM, 11.4 µM; 53 mM, 7.5 µM; 25 mM, 3.5 µM
AML 4	66200 µM / 7.7 µM = 8597	66.2 mM, 7.7 µM; 43 mM, 5 µM; 21.5 mM, 2.5 µM
AML 5	80900 µM / 8.3 µM = 9747	80.9 mM, 8.3 µM; 57.8 mM, 6 µM; 40.5 mM, 4 µM; 19.5 mM, 2 µM
AML 6	3700 µM / 2.6 µM = 1423	3.7 mM, 2.6 µM; 2.9 mM, 2 µM; 1.4 mM, 1 µM
AML 7	23800 µM / 17.4 µM = 1368	23.8 mM, 17.4 µM; 13.7 mM, 10 µM; 6.8 mM, 5 µM
AML 8	58900 µM / 23.8 µM = 2475	58.9 mM, 23.8 µM; 36.8 mM, 15 µM; 24.5 mM, 10 µM; 12.3 mM, 5 µM
AML 9	268000 µM / 13.5 µM = 19852	268 mM, 13.5 µM; 198.5 mM, 10 µM; 99.3 mM, 5 µM; 19.9 mM, 1 µM
AML 10	58800 µM / 2.23 µM = 26368	58.8 mM, 2.23 µM; 26.4 mM, 1 µM; 13.2 mM, 0.5 µM
AML 11	4800 µM / 14.5 µM = 331	4.8 mM, 14.5 µM; 2.4 mM, 7.25 µM; 1.2 mM, 3.6 µM
AML 12	56100 µM / 10.33 µM = 5431	56.1 mM, 10.3 µM; 28.1 mM, 5.2 µM; 14 mM, 2.6 µM; 7 mM, 1.3 µM
AML 13	200700 µM / 55.1 µM = 3642	200 mM, 55.1 µM; 100 mM, 27.6 µM; 50 mM, 13.8 µM
AML 14	43200 µM / 39.6 µM = 1091	43.2 mM, 39.6 µM; 21.6 mM, 19.8 µM;

		10.8 mM, 9.9 μM
AML 15	7950 μM / 70.2 μM = 113	8 mM, 70 μM; 4 mM, 35 μM;<br>2 mM, 17.5 μM; 1 mM, 8.75 μM
AML 16	95700 μM / 17 μM = 5629	95.7 mM, 17 μM; 47.9 mM, 8.5 μM;<br>23.9 mM, 4.25 μM; 12 mM, 2.13 μM
AML 17	81000 μM / 16.7 μM =4850	81 mM, 16.7 μM; 40.5 mM, 8.35 μM;<br>20.25 mM, 4.18 μM; 10.1 mM, 2.1 μM
AML 18	10300 μM / 4 μM = 2575	10.3 mM, 4 μM; 2.6 mM, 1 μM;<br>1.3 mM, 0.5 μM
AML 19	18200 μM / 0.55 μM = 33091	18.2 mM, 0.55 μM; 12.1 mM, 0.37 μM;<br>9.1 mM, 0.28 μM; 4.55 mM, 0.13 μM;<br>2.28 mM, 0.07 μM
AML 20	850 μM / 2.18 μM = 390	0.85 mM, 2.2 μM; 0.425 mM, 1.1 μM;<br>0.2 mM, 0.55 μM; 0.1 mM, 0.27 μM
AML 21	105300 μM / 22.8 μM = 4618	105.3 mM, 22.8 μM; 52.7 mM, 11.4 μM;<br>26.3 mM, 5.7 μM
CCCP (1st drug) / ABT-199 (2nd drug)		
OCI-AML2	15.63 μM / 0.177 μM = 88	15.63 μM, 0.177 μM; 10 μM, 0.113 μM;<br>5 μM, 0.06 μM; 1 μM, 0.0113 μM
PBMC	45.41 μM / 0.6 μM = 76	45.1 μM, 0.6 μM; 30 μM, 0.4 μM;<br>20 μM, 0.26 μM; 10 μM, 0.13 μM
2-DG (1st drug) / ABT-199 (2nd drug)		
OCI-AML2	16240 μM / 0.177 μM = 91751	16.24 mM, 0.177 μM; 13.8 mM, 0.15 μM;<br>9.2 mM, 0.1 μM; 1.8 mM, 0.02 μM
PBMC	106000 μM / 0.6 μM = 176667	106 mM, 0.6 μM; 53 mM, 0.3 μM;<br>26.5 mM, 0.15 μM; 10.6 mM, 0.06 μM
2-DG (1st drug) / ara-C (2nd drug)		
OCI-AML2	16240 μM / 77.8 μM = 209	16.2 mM, 77.8 μM; 8.1 mM, 38.9 μM;<br>4.1 mM, 19.5 μM; 2 mM, 9.7 μM
AML 11[1]	4800 μM / 53.4 μM = 90	4.8 mM, 53.4 μM; 2.4 mM, 26.7 μM;<br>1.2 mM, 13.4 μM
AML 13	200700 μM / 131.8 μM = 1523	200 mM, 131.8 μM; 100 mM, 69.1 μM;<br>50 mM, 34.5 μM
AML 14	43150 μM / 400 μM = 108	43.2 mM, 400 μM; 21.6 mM, 200 μM;<br>10.8 mM, 100 μM
AML 15	7950 μM / 50.4 μM = 158	8 mM, 50 μM; 4 mM, 25 μM;<br>2 mM, 12.5 μM; 1 mM, 6.25 μM
AML 20	850 μM / 141.2 μM = 6	0.85 mM, 141.2 μM; 0.425 mM, 70.6 μM;<br>0.2 mM, 35.3 μM; 0.1 mM, 17.7 μM

[1] Only primary AML samples, not resistant to ara-C (LD25 < 400 μM corresponding to 0.4% DMSO), have been treated with combination of ara-C and 2-DG.

Supplementary Table S9. Results of multiple linear regression: no association of pretreatment of the patients (n = 21) with mean combination indices of CCCP/2-DG.

Dependent variable[1]	B-coefficient	SE(B)	*p*-value
Before adjustment for age, gender, source of primary cells and % blast cells			
Mean combination index based on LD25, 50, 75 of the mixture CCCP/2DG	-0.085	0.118	0.481
After adjustment for age, gender, source of primary cells and % blast cells			
Mean combination index based on LD25, 50, 75 of the mixture CCCP/2DG	0.002	0.153	0.988

[1] With pretreatment (0 / 1) as an independent predictor

Supplementary Table S10. Correlations between mitochondrial parameters across untreated AML cells, healthy blood cells and SKOV3 cell line[1].

		Max OCR	ATP-linked OCR	Spare capacity	Coupling efficiency	LD25, CCCP	LD25, 2-DG	CI based on LD50 of the mixture	Mean CI based on LD25,50,75 of the mixture
Basal OCR	*r*	**0.915**	**0.975**	0.790	0.189	0.345	0.565	0.521	0.283
	p	**0.011**	**0.001**	0.061	0.719	0.500	0.243	0.289	0.587
Max OCR	*r*		**0.966**	**0.969**	0.477	0.463	0.644	0.579	0.397
	p		**0.002**	**0.001**	0.338	0.355	0.168	0.229	0.436
ATP-linked OCR	*r*			**0.888**	0.376	0.500	0.694	0.639	0.424
	p			**0.018**	0.463	0.312	0.126	0.172	0.402
Spare capacity	*r*				0.651	0.550	0.687	0.61	0.481
	p				0.161	0.258	0.132	0.199	0.335
Coupling efficiency	*r*					**0.840**	0.784	0.749	**0.821**
	p					**0.036**	0.065	0.087	**0.045**
LD25, CCCP	*r*						**0.969**	**0.907**	**0.893**
	p						**0.001**	**0.012**	**0.017**
LD25, 2-DG	*r*							**0.943**	**0.869**
	p							**0.005**	**0.025**
CI based on LD50 of the mixture	*r*								**0.960**
	p								**0.002**

[1] Correlations defined by two-tailed Pierson correlation coefficient *r*; *p* defines *p*-value. Significant correlations ($p<0.05$) are in bold.

Supplementary Table S11. Changes in mitochondrial bioenergetic parameters after treatment with mitocans (CCCP, DOX, LD50), glycolysis inhibitor (2-DG, LD50), or their combination (CCCP and 2-DG, LD25) in a panel of cells.

Drug	Bioenergetic parameter	MV-4-11	OCI-AML2	MOLM-13	THP-1	PBMC	SKOV3
CCCP, LD50	Basal OCR	$p < 0.0001$ **-74%**	$p < 0.0001$ **-92%**	$p < 0.0001$ **-89%**	$p < 0.0001$ **-96%**	NS	$p < 0.0001$ **-79%**
	Maximal OCR	$p < 0.0001$ **-67%**	$p < 0.0001$ **-96%**	$p < 0.0001$ **-96%**	$p < 0.0001$ **-98%**	$p < 0.0001$ **-88%**	$p < 0.0001$ **-88%**
	ATP-linked OCR	$p < 0.0001$ **-101%**	$p < 0.0001$ **-97%**	$p < 0.0001$ **-96%**	$p < 0.0001$ **-95%**	$p = 0.0020$ **-71%**	$p < 0.0001$ **-100%**
	Proton leak	NS	$p < 0.0001$ **-85%**	$p < 0.0001$ **-68%**	$p < 0.0001$ **-100%**	$p = 0.0034$ **+147%**	NS
	Spare capacity	$p = 0.0211$ **-64%**	$p < 0.0001$ **-100%**	$p < 0.0001$ **-100%**	$p < 0.0001$ **-100%**	$p < 0.0001$ **-105%**	$p < 0.0001$ **-96%**
	Coupling efficiency	$p < 0.0001$ **-116%**	$p = 0.0161$ **-31%**	$p = 0.0017$ **-19%**	$p < 0.0001$ **-45%**	$p = 0.0002$ **-105%**	$p < 0.0001$ **-129%**
	ECAR	NS	$p < 0.0047$ **-19%**	$p = 0.0001$ **-49%**	$p = 0.0004$ **-57%**	NS	NS
DOX, LD50	Basal OCR	$p < 0.0001$ **-54%**	$p = 0.0006$ **-51%**	$p < 0.0001$ **-68%**	$p = 0.0002$ **-81%**	$p = 0.0021$ **-35%**	NS
	Maximal OCR	$p = 0.0108$ **-30%**	$p = 0.0007$ **-62%**	$p < 0.0001$ **-77%**	$p < 0.0001$ **-77%**	$p = 0.0097$ **-52%**	NS
	ATP-linked OCR	$p < 0.0001$ **-65%**	$p = 0.0012$ **-69%**	$p = 0.0002$ **-84%**	$p = 0.0010$ **-89%**	$p = 0.0340$ **-38%**	NS
	Proton leak	NS	NS	NS	$p = 0.0090$ **-70%**	NS	NS
	Spare capacity	NS	$p = 0.0051$ **-77%**	$p = 0.0034$ **-83%**	$p = 0.0074$ **-74%**	$p = 0.0194$ **-55%**	$p = 0.0175$ **-47%**
	Coupling efficiency	$p = 0.0062$ **-30%**	$p = 0.0119$ **-55%**	NS	$p = 0.0157$ **-58%**	NS	NS
	ECAR	NS	NS	NS	$p = 0.0087$ **-48%**	$p = 0.0020$ **-34%**	NS
2-DG, LD50	Basal OCR	$p < 0.0001$ **-62%**	$p = 0.0047$ **-38%**	$p < 0.0001$ **-52%**	$p = 0.0020$ **-76%**	NS	$p < 0.0001$ **-47%**
	Maximal OCR	NS	NS	$p = 0.0017$ **-27%**	$p < 0.0001$ **-53%**	NS	$p = 0.0242$ **-52%**
	ATP-linked OCR	$p < 0.0001$ **-63%**	$p = 0.0270$ **-50%**	NS	NS	NS	$p < 0.0001$ **-71%**
	Proton leak	$p = 0.0076$ **-65%**	NS	$p = 0.0188$ **-35%**	$p = 0.0068$ **-63%**	NS	$p = 0.0004$ **+97%**
	Spare capacity	$p = 0.0017$ **+86%**	NS	NS	NS	NS	NS
	Coupling efficiency	NS	NS	NS	NS	NS	$p < 0.0001$ **-57%**
	ECAR	NS	$p < 0.0001$ **-37%**	$p = 0.0017$ **-47%**	$p = 0.0014$ **-56%**	$p < 0.0001$ **-60%**	NS
CCCP, LD25 + 2-DG, LD25	Basal OCR	$p < 0.0001$ **-81%**	$p = 0.0066$ **-42%**	$p < 0.0001$ **-83%**	NS	$p < 0.0001$ **-70%**	$p < 0.0001$ **-95%**
	Maximal OCR	$p < 0.0001$ **-52%**	$p < 0.0001$ **-72%**	$p < 0.0001$ **-89%**	$p < 0.0001$ **-61%**	$p < 0.0001$ **-94%**	$p < 0.0001$ **-96%**
	ATP-linked OCR	$p < 0.0001$ **-95%**	$p < 0.0001$ **-88%**	$p < 0.0001$ **-96%**	$p = 0.0103$ **-87%**	$p < 0.0001$ **-100%**	$p < 0.0001$ **-101%**

	Proton leak	*p* = 0.0056 **-67%**	NS	*p* = 0.0038 **-42%**	NS	NS	NS
	Spare capacity	NS	*p* < 0.0001 **-107%**	*p* < 0.0001 **-92%**	NS	*p* < 0.0001 **-99%**	*p* < 0.0001 **-98%**
	Coupling efficiency	*p* = 0.0003 **-72%**	*p* < 0.0001 **-80%**	*p* < 0.0001 **-67%**	*p* = 0.0005 **-64%**	*p* = 0.0016 **-85%**	*p* < 0.0001 **-121%**
	ECAR	NS	NS	*p* < 0.0001 **-58%**	NS	*p* < 0.0001 **-88%**	*p* = 0.0095 **-41%**

ANOVA with subsequent Dunn's or Fisher LSD test was used. Significant changes ($p<0.05$) are in bold.
